# Altered DNA methylation associated with a translocation linked to major mental illness

**DOI:** 10.1038/s41537-018-0047-7

**Published:** 2018-03-19

**Authors:** Daniel L. McCartney, Rosie M. Walker, Stewart W. Morris, Susan M. Anderson, Barbara J. Duff, Riccardo E. Marioni, J. Kirsty Millar, Shane E. McCarthy, Niamh M. Ryan, Stephen M. Lawrie, Andrew R. Watson, Douglas H. R. Blackwood, Pippa A. Thomson, Andrew M. McIntosh, W. Richard McCombie, David J. Porteous, Kathryn L. Evans

**Affiliations:** 1 Medical Genetics Section, Centre for Genomic and Experimental Medicine, Institute of Genetics and Molecular Medicine, Western General Hospital, University of Edinburgh, Crewe Road, Edinburgh, EH4 2XU UK; 2 Division of Psychiatry, Royal Edinburgh Hospital, University of Edinburgh, Edinburgh, EH10 5HF UK; 3 Department of Psychology, Centre for Cognitive Ageing and Cognitive Epidemiology, University of Edinburgh, 7 George Square, Edinburgh, EH8 9JZ UK; 4 Stanley Institute for Cognitive Genomics, Cold Spring Harbor Laboratory, Cold Spring Harbor, NY USA

## Abstract

Recent work has highlighted a possible role for altered epigenetic modifications, including differential DNA methylation, in susceptibility to psychiatric illness. Here, we investigate blood-based DNA methylation in a large family where a balanced translocation between chromosomes 1 and 11 shows genome-wide significant linkage to psychiatric illness. Genome-wide DNA methylation was profiled in whole-blood-derived DNA from 41 individuals using the Infinium HumanMethylation450 BeadChip (Illumina Inc., San Diego, CA). We found significant differences in DNA methylation when translocation carriers (*n* = 17) were compared to related non-carriers (*n* = 24) at 13 loci. All but one of the 13 significant differentially methylated positions (DMPs) mapped to the regions surrounding the translocation breakpoints. Methylation levels of five DMPs were associated with genotype at SNPs in linkage disequilibrium with the translocation. Two of the five genes harbouring significant DMPs, *DISC1* and *DUSP10*, have been previously shown to be differentially methylated in schizophrenia. Gene Ontology analysis revealed enrichment for terms relating to neuronal function and neurodevelopment among the genes harbouring the most significant DMPs. Differentially methylated region (DMR) analysis highlighted a number of genes from the MHC region, which has been implicated in psychiatric illness previously through genetic studies. We show that inheritance of a translocation linked to major mental illness is associated with differential DNA methylation at loci implicated in neuronal development/function and in psychiatric illness. As genomic rearrangements are over-represented in individuals with psychiatric illness, such analyses may be valuable more widely in the study of these conditions.

## Altered DNA methylation implicated in mental illness

Kathryn Evans and colleagues at the University of Edinburgh, UK, analysed the DNA of 41 members of a family that includes 17 people with major mental illness (schizophrenia, major depressive disorder, bipolar disorder and other conditions) associated with the translocation of genetic material between chromosomes 1 and 11. The analyses showed differences between translocation carriers and non-carriers in the levels of methyl groups attached to their DNA in the regions of the genome surrounding the translocation, and elsewhere in genes associated with the immune system and with nervous system development and function. Further work is needed to confirm if and investigate how these differences in DNA methylation are related to psychiatric illness.

## Introduction

We previously reported a single, large family where a balanced translocation between chromosomes 1 and 11 [t(1;11)] shows genome-wide significant linkage for SZ, rMDD and BD.^1,2^ On chromosome 1, the translocation disrupts the protein coding gene *Disrupted in schizophrenia 1* (*DISC1*) and the antisense non-coding gene *Disrupted in Schizophrenia 2* (*DISC2*). On chromosome 11, the translocation disrupts the non-coding gene *DISC1 Fusion Partner 1* (*DISC1FP1*).^3,4^ *DISC1* is expressed in the brain throughout development and adulthood, where it plays a role in multiple processes including neurogenesis and neuronal migration, integration, maturation and signalling.^5^ Lymphoblastoid cell lines from carriers of the translocation show a ~50% reduction in DISC1 protein expression, implicating haploinsufficiency as a potential pathogenic mechanism.^3^

It is known that chromosomal translocations can be associated with altered chromatin organisation coupled with transcriptional deregulation, and that these effects can extend a considerable distance beyond the breakpoint locations.^6–8^ DNA methylation is an important key component of chromatin structure that is associated with the regulation of gene expression.^9^ Altered methylation is present in individuals with SZ and BD, both in MZ twins who are discordant for illness^10^ and in case/control comparisons of each of these illnesses.^11–13^ In light of this evidence, we hypothesised that investigating DNA methylation in the t(1;11) pedigree may further our understanding of the aetiology of illness in this family.

To assess whether altered DNA methylation exists and, therefore, might contribute to illness in t(1;11) carriers, we profiled genome-wide methylation in whole-blood-extracted DNA from 17 translocation carriers and 24 family members without the translocation (non-carriers). We compared methylation levels in carriers to those in non-carriers, to address the hypothesis that inheritance of the translocated chromosomes is associated with differential DNA methylation.

## Results

### Assessment of differential methylation between t(1;11) carriers and non-carriers

**Fig. 1:**
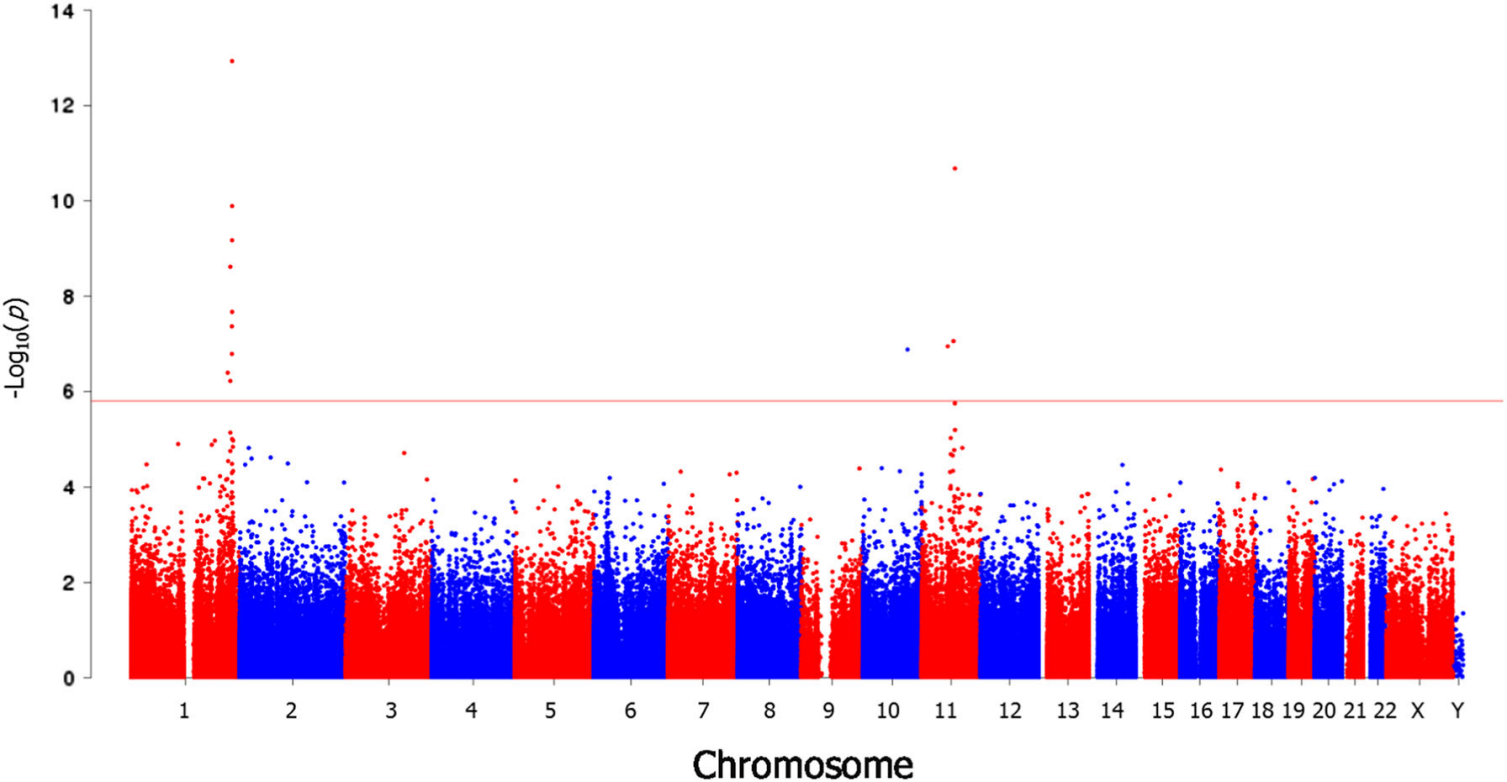
Manhattan plot for DNA methylation comparison between t(1;11) carriers and non-carriers. Figure shows –log10 *p*-values for differential methylation between t(1;11) carriers and non-carriers (*y-*axis) plotted against chromosomal position (*x-*axis). The horizontal red line represents the –log10 *p-*value threshold for genome-wide significance (FDR *q* = 0.05)

**Fig. 2:**
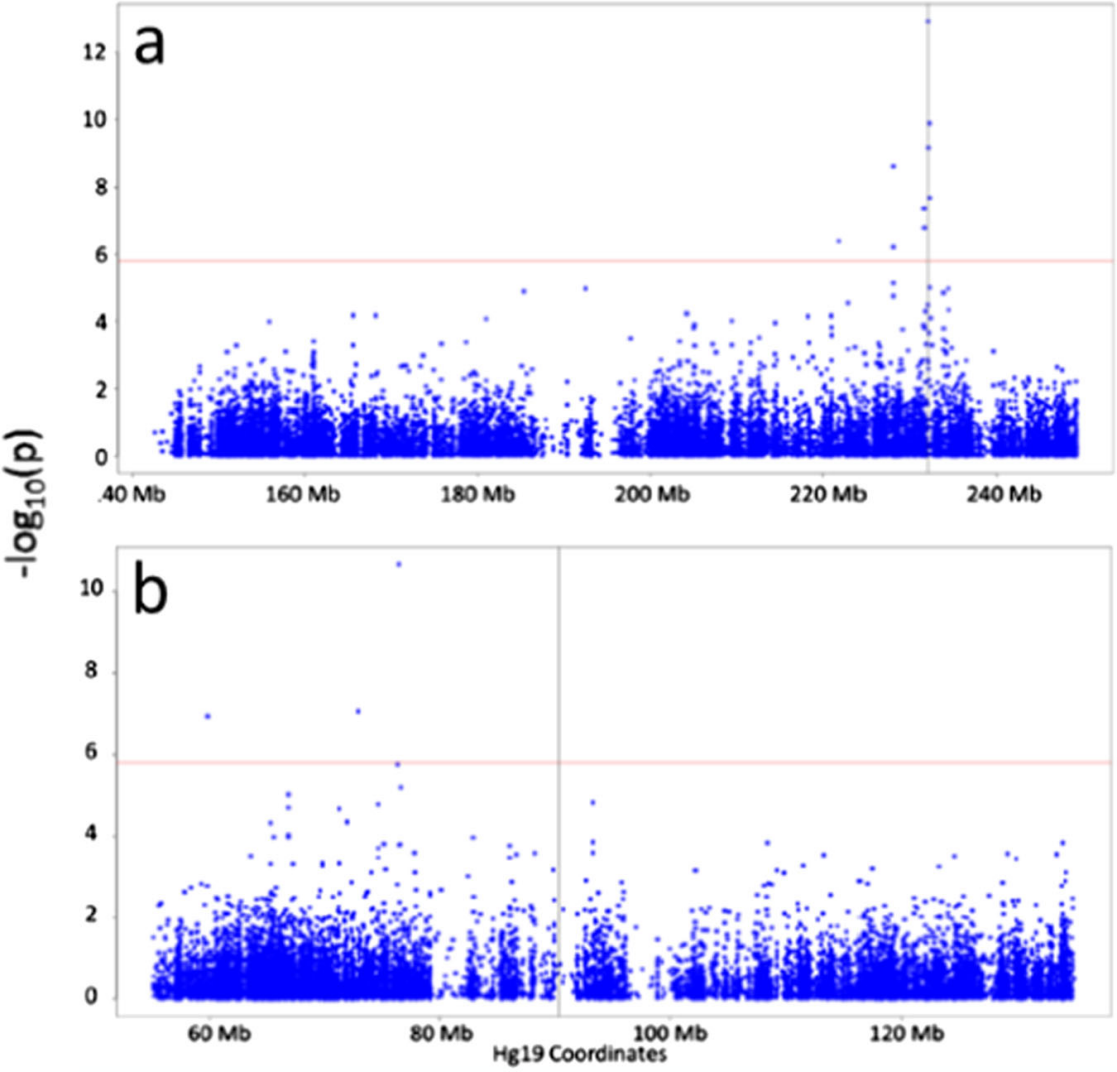
Manhattan plots for DNA methylation comparison between t(1;11) carriers and non-carriers at translocation breakpoint regions. Figure shows –log10 *p-*values for differential methylation between t(1;11) carriers and non-carriers (*y-*axis) plotted against chromosomal position (*x-*axis) for chromosome 1 (**a**) and chromosome 11 (**b**). The horizontal red line represents the –log10 *p-*value threshold for genome-wide significance (FDR *q* = 0.05). The vertical black line denotes the translocation breakpoint

DNA methylation was compared between 17 t(1;11) carriers and 24 non-carriers by linear regression, fitting age, gender and eight significant surrogate variables identified as covariates. Prior to analysis, we assessed for confounding between translocation carrier status and age, gender and estimated cellular proportions. Unpaired *t*-tests and Fisher’s exact tests were performed to identify whether age and gender, respectively, were significantly associated with group status. A significant between-group difference in age was observed between t(1;11) carriers and non-carriers, with carriers being older (*p* = 0.045; Supplementary Table 1). No significant differences were observed between gender (*p* = 1; Supplementary Table 2). Differences in cell composition were assessed for statistical significance using a Student’s *t*-test. No significant between-group differences were observed (*p* ≥ 0.407; Supplementary Table 3). Significant differential methylation was observed at 13 loci (false discovery rate (FDR) *q* < 0.05; Fig. 1, Table 1). Four of these sites were in the *DISC1* gene, three mapping to the gene body and one to the 3′ untranslated region (3′UTR). With the exception of one site on chromosome 10 (cg24508974), all differentially methylated sites mapped to either chromosome 1 or 11. Of the 12 sites on chromosomes 1 and 11, all but one were hypomethylated in t(1;11) carriers. The most distal differentially methylated sites were situated approximately 10 Mb and 31 Mb from the translocation breakpoints on chromosomes 1 and 11, respectively (Fig. 2; Table 1). The most significant single differentially methylated position (DMP) (cg09186051) was in intron 9 of *DISC1* and intron 2 of *DISC2* (JK Millar, personal communication), approximately 30 kb telomeric of the chromosome 1 breakpoint.

**Table 1:** Significantly differentially methylated positions between t(1;11) carriers and non-carriers

Probe ID	Hg19 coordinates	Gene	Beta difference	Fold-change	*t*	*p*-value	*q*-value
cg09186051	Chr1:231981906	*DISC1*;T*SNAX-DISC1*	−0.07	−1.31	−11.81	1.17 × 10^−13^	5.20 × 10^−8^
cg26728851	Chr11:76430375	*GUCY2E*	−0.03	−1.63	−9.72	2.09 × 10^−11^	4.64 × 10^−6^
cg15157974	Chr1:232144702	*DISC1*;*TSNAX-DISC1*	−0.04	−1.27	−9.04	1.30 × 10^−10^	1.92 × 10^−5^
cg05656812	Chr1:232021560	*DISC1*;*TSNAX-DISC1*	−0.06	−1.33	−8.44	6.73 × 10^−10^	7.46 × 10^−5^
cg06928246	Chr1:227974645	NA	−0.07	−1.56	−7.99	2.41 × 10^−09^	0.0002
cg16177633	Chr1:232172585	*DISC1*;*TSNAX-DISC1*	−0.03	−1.20	−7.23	2.13 × 10^−08^	0.0016
cg18815120	Chr1:231512676	*EGLN1*	−0.12	−2.10	−6.99	4.29 × 10^−08^	0.003
cg25899154	Chr11:72897143	NA	−0.07	−1.28	−6.75	8.73 × 10^−08^	0.005
cg02771260	Chr11:59836817	*MS4A3*	−0.13	−1.79	−6.66	1.13 × 10^−07^	0.006
cg24508974	Chr10:103330391	NA	0.01	1.17	6.61	1.32 × 10^−07^	0.006
cg21875980	Chr1:231553510	*EGLN1*	0.06	1.40	6.54	1.64 × 10^−07^	0.007
cg26355502	Chr1:221916303	*DUSP10*	−0.01	−1.30	−6.24	4.05 × 10^−07^	0.01
cg00965168	Chr1:227974541	NA	−0.05	−1.42	−6.10	6.03 × 10^−07^	0.02

Table summarises significantly differentially methylated sites between t(1;11) carriers and non-carriers (FDR *q* ≤ 0.05). In order of column appearance are probe identifiers, Hg19 genomic coordinates, UCSC reference gene names (“NA” denotes intergenic regions), between-group difference in mean beta value, fold-change between groups, moderated *t*-statistic, *p*-value for differential methylation and FDR-adjusted *p*-value (*q*-value)

### Gene Ontology analysis

Although only 13 probes remained significant at a false discovery rate of 5%, it is possible that the multiple non-significant differences in methylation affecting other genomic loci affect common biological pathways and processes. In order to establish whether the genes most affected by differential methylation were enriched for particular biological functions, Gene Ontology (GO) analysis was performed on a list of genes ranked by uncorrected *p-*value from the DMP analysis (*n* = 20,752). Among the top-ranked genes, 62 GO terms showed significant enrichment (*q* ≤ 0.05; Supplementary Table 4). These included several neurologically relevant terms, for example, the most significant was “neuron projection” (*q* = 3.72 × 10^−6^, *n* = 131 genes).

### Assessment of differentially methylated regions

**Fig. 3:**
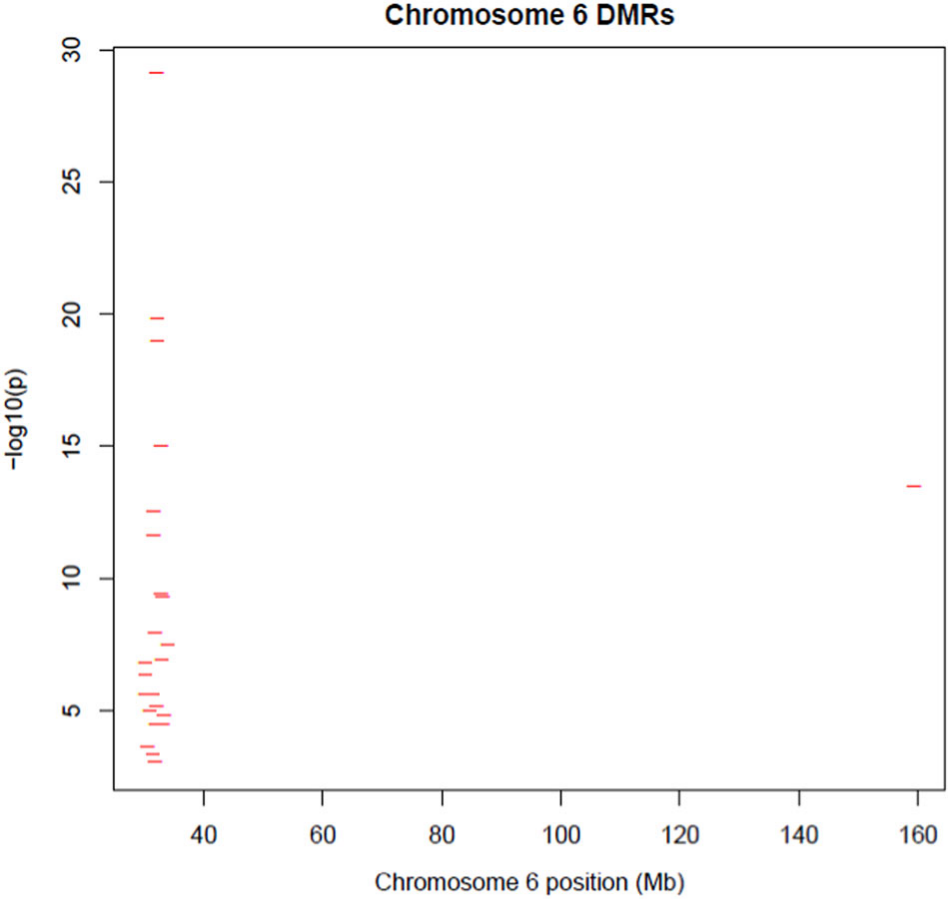
Differentially methylated regions (DMRs) on chromosome 6. Figure shows –log10 *p-*values for differentially methylated regions identified in t(1;11) carriers (*y-*axis) plotted against chromosomal position in Mb (*x-*axis) for chromosome 6

Nominally significant probes (*p* ≤ 0.05; *n* = 24,149) were included in the differentially methylated region (DMR) analysis, identifying 123 DMRs (*q* ≤ 0.05; Supplementary Table 5). The most significant DMR was located in *TNXB* (*p* = 2.5 × 10^−13^). Overlap was assessed between genes containing DMRs (*n* = 94) and findings of genome-wide association studies (GWASs) of SZ, BD and major depressive disorder.^14–20^ Twenty-two of the DMRs identified were within the major histocompatibility complex (MHC; Fig. 3), which has been implicated in the pathogenesis of SZ through a large-scale GWAS.^16^ In addition, we identified DMRs within two additional genes (*IGSF9B*, *CNTN4*) that showed genome-wide association with SZ in the same study. No overlap was observed between DMR genes and GWAS findings in BD or MDD.^14,15,17–21^ A graphical summary of all DMRs identified is available in Supplementary File 2.

### Assessment of the role of genetic variation in regulating DNA methylation

A recent study^22^ identified a large number of loci at which methylation level was associated with genotype at a single-nucleotide polymorphism (SNP) either in *cis* or in *trans* with the methylation site (methylation quantitative trait loci; meQTL). Seven of our 13 significant DMPs (*q* < 0.05) were associated with a meQTL observed in this study.^22^ We attempted to replicate the findings for these seven probes and found that five were significant meQTLs in our study (*p* < 0.05 Table 2). All five loci were located on chromosome 1 or 11 and were significantly associated with the translocation (*p* < 0.05; Table 2).

**Table 2:** Summary of meQTLs reported to regulate DNA methylation at differentially methylated loci identified between t(1;11) carriers and non-carriers

Reported meQTL	Probe ID	Distance between probe and SNP	Probe gene	meQTL gene	meQTL *p*-value
rs2486729*	cg18815120	23 kb	*EGLN1*	*EGLN1*	2.02 × 10^−16^
rs17154511*	cg02771260	11 kb	*MS4A3*	*MS4A3*	8.14 × 10^−11^
rs10899287*	cg26728851	84 kb	*GUCY2E*	Intergenic	0.0002
rs545937*	cg21875980	10 kb	*EGLN1*	*EGLN1*	3.53 × 10^−5^
rs4366301*	cg16177633	366 kb	*DISC1*;*TSNAX-DISC1*	*DISC1*;*TSNAX-DISC1*	0.0009
rs6541279*	cg15157974	391 kb	*DISC1*;*TSNAX-DISC1*	*TSNAX-DISC1*	0.737
rs9419922	cg24508974	50 kb	Intergenic	Intergenic	0.548

From left to right, columns show the probe identifier, the corresponding probe’s associated gene, previously reported^22^ meQTL, the gene containing the meQTL, and the *p*-value for the relationship between DNA methylation and meQTL genotype, adjusting for age and sex. meQTLs accompanied by an asterisk (*) denote those that are significantly associated with t(1,11) carrier status (*p* ≤ 0.05)

## Discussion

We characterised genome-wide DNA methylation in whole-blood-derived DNA from 41 members of a family in which a translocation, t(1;11), segregates with major mental illness, including SZ, rMDD and BD. The overlapping genetic architecture of these disorders has previously been established through the identification of disease-associated variants,^15^ as well as transcriptomic^23^ and methylomic^11^ case–control differences. There are multiple mechanisms by which the translocation might confer risk for psychiatric illness; our goal was to investigate whether it was associated with altered DNA methylation, which has been previously implicated in psychiatric illness.

Comparison of DNA methylation in t(1;11) carriers and non-carriers identified 13 significant DMPs. All but one mapped to the regions surrounding the translocation breakpoints: nine occurred within five genes, while the remainder were intergenic. Four significant DMPs mapped to the *DISC1* gene, which is interrupted by the translocation and has been implicated in neurodevelopment, cognitive function and susceptibility to psychiatric illness.^24–27^ Differential methylation in two of the five genes, *DISC1* and *DUSP10*, was also observed in a recent study of differential methylation associated with SZ^13^ although it should be noted that different probes were implicated. Two DMPs identified here mapped to the *EGLN1* gene, which encodes prolyl hydroxylase domain-containing protein 2 (PHD2). PHD2 regulates the transcription factor HIF-1α, the master transcriptional regulator of the cellular response to hypoxia,^28^ which is an obstetric and developmental risk factor for SZ.^29–31^

Only a subset of the CpG sites adjacent to the breakpoints showed significant differential methylation. There are a number of possible reasons for this observation, for example: genetic control of methylation at specific CpG sites by meQTL in linkage disequilibrium with the translocation; tissue-specific differential methylation patterns and/or lack of power. With regard to meQTL, 7 of the 13 significant DMPs were previously reported to be influenced by an meQTL in a study of lymphocyte DNA methylation.^22^ We were able to replicate these findings for five of these probes. The failure to replicate the remainder may be attributable to limited statistical power (due to a small number of homozygote carriers of the minor allele of the meQTL).

Using GO analysis, we investigated whether the genes harbouring the most significantly differentially methylated loci were enriched for particular biological processes. This pointed to the possibility of the translocation conferring an effect upon neurodevelopment in t(1;11) carriers. This is in keeping with findings of structural and functional differences in both affected and unaffected carriers of the translocation^1,2,32^ and of neurodevelopmental abnormalities observed in SZ more widely.^33^

Between-locus correlation in DNA methylation is common at neighbouring methylation loci. Differences in methylation across a number of adjacent sites (even if not statistically significant individually) may together confer a biologically meaningful effect.^34^ We, therefore, carried out DMR analyses and identified one gene, *TNXB*, as being particularly noteworthy. This gene contained the top *p*-value-ranked DMR (51 probes spanning approximately 1.6 kb) along with a second region (325 bp, comprising eight probes). *TNXB* encodes Tenascin X, an extracellular glycoprotein, predominantly expressed in connective tissues. *TNXB*, in common with a number of the genes identified in this study, is located within the extended MHC region, which was identified as the most significant locus in a recent GWAS of SZ.^16^ Evidence for altered immune function in psychiatric illness is long established.^35^

Two additional DMRs were identified within genes associated with SZ at the genome-wide significant level by the SZ Working Group of the Psychiatric Genomics Consortium (PGC).^16^ These were within the genes *IGSF9B* and *CNTN4*, both of which function as cell adhesion molecules. Two large-scale epigenome-wide association studies of SZ have recently been reported.^12,13^ These studies reported significant differential methylation in *RPTOR*: a gene in which we identified a DMR. *RPTOR* is a key component of mTOR signalling, which has been implicated in synaptic plasticity.^36^

None of the DMRs identified contained any of the 13 significant DMPs identified when t(1;11) carriers were compared to non-carriers. Of these 13 DMPs, 7 could not contribute to DMRs as the nearest adjacent probe falls outside the maximum radius for the DMR-calling “lasso”. This highlights a limitation of DMR analysis: the identification of DMRs requires several parameters, such as the minimum number of probes required to form a DMR and the distance permissible between these probes, to be set. As there is a dearth of experimental evidence linking the selection of DMR parameters to the identification of biologically meaningful DMRs, parameter setting is somewhat arbitrary. It is interesting to note, for example, that three adjacent probes mapping to *DISC1* (and *DISC2*) met the criteria for significant differential methylation, but were spaced slightly too far apart to be called as a DMR. Moreover, in the majority of cases, the Infinium HumanMethylation450 BeadChip does not interrogate all adjacent CpGs that could constitute a DMR. This issue could be addressed in the future by a targeted follow-up approach (such as bisulfite sequencing). It is important to note that all translocation carriers in this analysis have a psychiatric diagnosis. While the translocation has been significantly linked to psychiatric illness, it is not possible to determine whether the DMR findings are directly linked to the translocation or rather related to illness in these individuals. The same argument could be made for the DMP findings. However, the proximity of the strongest signals to the regions surrounding the breakpoints are consistent with a role for the translocation in the DMP analysis.

A limitation of this study is the use of blood-derived (as opposed to brain-derived) DNA. Of relevance to this point, others have reported that only 4.1% of 450k methylation array probes were strongly correlated between blood and neocortex biopsied at the point of surgical intervention for pharmaco-resistant epilepsy.^37^ However, others report meQTLs to be consistently detected across tissue types.^38^ This suggests that, should the differences in methylation observed reflect the effects of variants in linkage disequilibrium with the translocation, these methylation differences might be consistently observed across tissue types. A further caveat is the fact that at the time of blood draw some individuals in the study will have been taking medication that may have had an impact on DNA methylation. Between-group differences in cell composition are a common concern in studies involving blood-derived samples. However, we expect that the eight variables identified by surrogate variable analysis should account for cellular heterogeneity, among other unmodeled factors.^39^ It is also important to acknowledge the limitations in statistical power posed by a small sample. Although relatively small in terms of sample size, the family-based design of the current study should reduce the levels of genetic heterogeneity observed in larger unrelated case–control comparisons, thus increasing statistical power.

To conclude, we have found evidence for altered DNA methylation in carriers of a translocation that is linked to an increased risk of schizophrenia and other major psychiatric illnesses.^2^ Further work is, however, required to determine the cause(s) and consequences of these differences in DNA methylation, and thus whether and how they might relate to the adverse effects of inheritance of the translocation. Given the existence of other highly penetrant structural variants in psychiatric illness, it will be of interest to determine whether this is a fruitful model for future studies of these conditions.

## Methods

### Sample information

Genome-wide DNA methylation was profiled in 41 members of the t(1;11) family.^2^ Of these, 17 were carriers of the t(1;11) translocation and 24 were non-carriers (t(1;11) carriers: 47% males, mean age = 49 years; t(1;11) non-carriers: 50% males, mean age = 37 years). Unpaired *t*-tests and Fisher’s exact tests were performed to identify whether age and gender, respectively, were significantly associated with group status. Diagnostic information is provided in Supplementary Table 6.

### Study approval

The study was approved by the Multicentre Research Ethics Committee for Scotland (09/MRE00/81). A detailed description of the study was given and written informed consent was obtained from all individuals before participation.

### Sample preparation

Whole-blood-derived genomic DNA (500 ng) was treated with sodium bisulphite using the EZ-96 DNA Methylation Kit (Zymo Research, Irvine, CA), following the manufacturer’s instructions. Samples were analysed using the Infinium HumanMethylation450 BeadChip (Illumina Inc., San Diego, CA) at the Wellcome Trust Clinical Research Facility (WTCRF), Western General Hospital, Edinburgh, UK. Samples were assigned to chips such that, as far as possible, group and gender were balanced across chips.

### Quality control, data pre-processing and normalisation

Raw intensity (.idat) files were read into R using the *minfi* package,^40^ which was used to perform initial quality control assessments. Initial quality control assessments were performed by inspecting plots of signal from the internal control probes to assess the success of each stage of the experimental process, for example, bisulphite conversion and probe hybridisation. Signal from these control probes was acceptable for all samples. Prior to normalisation, the data were filtered to remove poorly performing probes as follows: Firstly, 30,969 probes that have been predicted to hybridise to multiple genomic regions were removed.^41^ Next, 10,548 probes affected by genetic variation at the target CpG or the site of single-base extension (for Type I probes) were excluded.^41^ Finally, 799 probes with more than five samples with a bead count of less than three and/or ≥1% samples with a detection *p*-value of >0.05 were removed. The criteria for sample removal were: (i) if a sample failed any of the quality control assessments carried out in *minfi*; or (ii) ≥1% of sites had a detection *p*-value of >0.05 in a given sample. No samples met the quality control criteria for removal and the final data set consisted of 443,196 probes and 41 samples. Thirteen normalisation methods were compared based on their ability to reduce technical variation, using the R package *wateRmelon*,^42,43^ identifying the dasen method as the normalisation method that best reduced technical variation in the data set.^42^ The data were then normalised using this method, which involves adjusting the background difference between Type I and Type II assays (by adding the offset between Type I and II probe intensities to Type I intensities). Between-array quantile normalisation was then performed for the methylated and unmethylated signal intensities separately (Type I and Type II assays normalised separately) and methylation beta values were calculated. Prior to downstream analyses, *M*-values, defined as *M* = log2((*M* + 100)/(*U* + 100)), where *M* represents the methylated signal intensity and *U* represents the unmethylated signal intensity, were calculated for the normalised data.

### Assessment of between-group differences in whole-blood cellular composition

As variation in the cellular proportions of whole blood has the potential to confound between-group comparisons of DNA methylation, we investigated whether predicted cell proportions differed between t(1;11) carriers and non-carriers. Estimated cell counts for B-lymphocytes, granulocytes, monocytes, natural killer cells, CD4+ T-lymphocytes and CD8+ T-lymphocytes were produced using the *minfi* estimateCellCounts() function, which implements Jaffe and Irizarry’s modified version of Houseman’s algorithm.^39,44^

### Surrogate variable analysis

In order to account for latent, unmodelled sources of variation with potentially confounding effects, surrogate variables were estimated and fitted as covariates in the DMP analysis.^45^ Eight significant surrogate variables were identified using the “be” method in the R package *sva*, after specifying age and gender as covariates,^46^ and fitted as covariates in the final regression model.

### Assessment of DMPs

DMPs between groups were identified by linear regression using the R package *limma*,^47^ adjusting for age, gender and significant surrogate variables identified using the *sva* package.^45^ Summary statistics were computed using *limma*’s eBayes() function,^47^ in which estimated probe-wise variances were adjusted towards a common value. Normality of the data was assessed by visual inspection of raw *p*-values in a quantile–quantile plot (Supplementary Figure 18). Multiple testing correction was implemented using the Benjamini–Hochberg FDR,^48^ with a *q*-value of ≤0.05 deemed to represent genome-wide significance.

### Assessment of DMRs

DMRs were assessed using the champ.lasso() function available in the *ChAMP* package.^49,50^ Probes with an uncorrected *p*-value of ≤0.05 in the DMP analyses were submitted for DMR analysis. A DMR was defined as a set of at least three contiguous nominally significant differentially methylated positions located within a defined “lasso radius”, which was set at the function’s default of a maximum of 2 kb. To account for the probe spacing bias on the 450k array, the value of this “radius” was scaled, using default parameters based on the genomic feature (e.g., CpG island, intergenic region) associated with an underlying probe. The default minimum DMR separation threshold of 1 kb was also applied, below which adjacent DMRs were merged.

### GO analysis

Ranked list (by *p*-value) GO analysis was performed using GOrilla.^51^ In common with other GO and pathway methods, GOrilla accepts each gene identifier only once.^52^ Therefore, where genes were represented by multiple probes, the lowest DMP *p*-value for any associated probe was used to rank the gene for GO analysis. GOrilla utilises a “flexible threshold” to determine enrichment at the top of the ranked list to define a “target set” at the top of the list and a “background set” comprising all other genes in the list. A hypergeometric test is performed to assign a *p*-value to each GO category and a Benjamini–Hochberg FDR is then calculated to reflect the number of GO categories assessed. GO categories with an FDR *q*-value ≤0.05 were considered statistically significant.

### Assessment of meQTLs

A list of previously reported meQTLs and their associated CpGs^22^ was interrogated to establish whether methylation at any CpG site showing significant differential methylation in this study (*q* ≤ 0.05) was likely to be regulated by an meQTL. For each meQTL, genotype information was obtained from whole-genome sequence data for the family (Ryan et al., submitted).^53^ For those CpGs previously identified as being regulated by a meQTL,^22^ linear regression was performed to assess the relationship between minor allele count at the SNP concerned and methylation *M*-values. A *p*-value of ≤0.05 was considered statistically significant.

### Data availability

The code used in this analysis (including version information) can be accessed by contacting the corresponding author.

## Electronic supplementary material

Supplementary Tables

Supplementary Figures
