# Supplementary material for: Altered DNA methylation associated with a translocation linked to major mental illness
Source: NPJ Schizophr. 2018 Mar 19;4:5. doi: 10.1038/s41537-018-0047-7 (PMC5859082; doi:10.1038/s41537-018-0047-7)
Supplement: Supplementary file 1 — Supplementary Tables [file 41537_2018_47_MOESM1_ESM.pdf]

	Mean Age	*p - value*
t(1;11) Carriers	49	0.045
t(1;11) Non-carriers	37	

Supplementary Table 1 Comparison of ages between t(1;11) carriers and non-carriers

Shown are the mean ages of t(1;11) carriers and non-carriers, and unpaired t-test *p*-value comparing between-group age.

	Males	*Females*	*p-value*
t(1;11) Carriers	8	9	1
t(1;11) Non-carriers	12	12	

Supplementary Table 2 Comparison of gender between t(1;11) carriers and non-carriers

Shown are the counts for male and females amongst t(1;11) carriers and non-carriers, and Fisher's exact *p*-value.

	$CD8^+$ T-Cells	*$CD4^+$ T-Cells*	*Natural Killer Cells*	*B-Cells*	*Monocytes*	*Granulocytes*
Mean proportion in t(1;11) Carriers	0.05	0.15	0.06	0.06	0.07	0.62
Mean proportion in t(1;11) Non-carriers	0.05	0.16	0.06	0.05	0.07	0.62
p- value	0.7	0.58	0.5	0.41	0.85	0.87

Supplementary Table 3 Comparison of estimated cellular composition of whole blood between (1;11) carriers and non-carriers

Shown are the mean estimated proportions for blood cell types in t(1;11) carriers and non-carriers, and unpaired t-test *p*-values for each cell type.

GO Term	*Description*	*Class*	*Enrichment*	*p-value*	*q-value*
GO:0043005	neuron projection	Component	1.73	2.29×10^{-9}	3.72×10^{-6}
GO:0042995	cell projection	Component	1.44	3.63×10^{-7}	0.0003
GO:0030425	dendrite	Component	1.43	2.10×10^{-6}	0.0009
GO:0009653	anatomical structure morphogenesis	Process	1.5	6.49×10^{-8}	0.0009
GO:0097458	neuron part	Component	1.96	1.93×10^{-6}	0.001
GO:0009987	cellular process	Process	1.33	5.98×10^{-7}	0.002
GO:0048562	embryonic organ morphogenesis	Process	4.36	4.93×10^{-7}	0.0022
GO:0048856	anatomical structure development	Process	1.07	4.14×10^{-7}	0.0028
GO:0048731	system development	Process	1.72	1.04×10^{-6}	0.0028
GO:0030424	axon	Component	1.12	3.05×10^{-5}	0.0083
GO:0044444	cytoplasmic part	Component	4.26	2.75×10^{-5}	0.0089
GO:0032502	developmental process	Process	1.3	6.12×10^{-6}	0.0093
GO:0010646	regulation of cell communication	Process	1.25	4.91×10^{-6}	0.0096
GO:0044763	single-organism cellular process	Process	1.1	5.69×10^{-6}	0.0097
GO:0050793	regulation of developmental process	Process	1.21	4.54×10^{-6}	0.01
GO:0048812	neuron projection morphogenesis	Process	2.26	8.99×10^{-6}	0.012
GO:0009887	organ morphogenesis	Process	2.15	8.55×10^{-6}	0.017
GO:0051960	regulation of nervous system development	Process	1.25	1.29×10^{-5}	0.014
GO:0023051	regulation of signaling	Process	1.54	1.52×10^{-5}	0.014
GO:0051094	positive regulation of developmental process	Process	1.41	1.43×10^{-5}	0.014
GO:0048583	regulation of response to stimulus	Process	1.25	1.24×10^{-5}	0.014
GO:0050807	regulation of synapse organisation	Process	6.99	2.31×10^{-5}	0.02
GO:0044424	intracellular part	Component	1.07	8.74×10^{-5}	0.02
GO:0070588	calcium ion transmembrane transport	Process	1.21	4.33×10^{-5}	0.021
GO:0051962	positive regulation of nervous system development	Process	1.93	2.97×10^{-5}	0.021
GO:0044708	single-organism behavior	Process	1.68	4.57×10^{-5}	0.022
GO:2000026	regulation of multicellular organismal development	Process	1.38	3.19×10^{-5}	0.022
GO:0045595	regulation of cell differentiation	Process	1.56	3.52×10^{-5}	0.022
GO:2001030	negative regulation of cellular glucuronidation	Process	1.34	4.33×10^{-5}	0.022
GO:0048858	cell projection morphogenesis	Process	1.98	2.90×10^{-5}	0.022
GO:0009888	tissue development	Process	1.63	3.75×10^{-5}	0.022
GO:0050767	regulation of neurogenesis	Process	25.57	3.46×10^{-5}	0.023q
GO:0031344	regulation of cell projection organisation	Process	25.57	3.98×10^{-5}	0.023

GO:1904224	negative regulation of glucuronosyltransferase activity	Process	25.57	4.33×10^{-5}	0.023
GO:0044767	single-organism developmental process	Process	2.35	2.87×10^{-5}	0.023
GO:1904223	regulation of glucuronosyltransferase activity	Process	1.79	4.33×10^{-5}	0.024
GO:0021852	pyramidal neuron migration	Process	17,640	5.67×10^{-5}	0.026
GO:0051239	regulation of multicellular organismal process	Process	1.29	6.25×10^{-5}	0.028
GO:0032990	cell part morphogenesis	Process	1.84	6.46×10^{-5}	0.028
GO:0003197	endocardial cushion development	Process	1.45	7.97×10^{-5}	0.028
GO:2001029	regulation of cellular glucuronidation	Process	2.01	7.94×10^{-5}	0.029
GO:0045597	positive regulation of cell differentiation	Process	1.75	7.08×10^{-5}	0.029
GO:0052697	xenobiotic glucuronidation	Process	46.22	7.94×10^{-5}	0.029
GO:2000467	positive regulation of glycogen (starch) synthase activity	Process	22.72	7.90×10^{-5}	0.03
GO:0046578	regulation of Ras protein signal transduction	Process	22.72	7.55×10^{-5}	0.03
GO:0048598	embryonic morphogenesis	Process	21.83	7.86×10^{-5}	0.031
GO:0051240	positive regulation of multicellular organismal process	Process	1.35	9.01×10^{-5}	0.031
GO:0048513	organ development	Process	1.43	0.0001	0.034
GO:0034702	ion channel complex	Component	1.81	0.0002	0.035
GO:0072300	positive regulation of metanephric glomerulus development	Process	213.82	0.0001	0.037
GO:0005783	endoplasmic reticulum	Component	5.76	0.0003	0.041
GO:0005768	endosome	Component	1.64	0.0003	0.042
GO:0014069	postsynaptic density	Component	35.18	0.0003	0.043
GO:0048167	regulation of synaptic plasticity	Process	1.79	0.0001	0.044
GO:0044699	single-organism process	Process	1.07	0.0001	0.044
GO:0044216	other organism cell	Component	5.29	0.0003	0.044
GO:0044297	cell body	Component	1.43	0.0002	0.045
GO:0050804	modulation of synaptic transmission	Process	2.5	0.0001	0.045
GO:0043524	negative regulation of neuron apoptotic process	Process	2.63	0.0002	0.046
GO:0033387	putrescine biosynthetic process from ornithine	Process	22.88	0.0002	0.047
GO:0031225	anchored component of membrane	Component	1.38	0.0004	0.048
GO:0044463	cell projection part	Component	4.34	0.0004	0.049

Supplementary Table 4 Summary of GO terms enriched amongst differentially methylated genes in t(1;11) carriers

For each GO term, Supplementary Table ummarises the GO identifier, the GO description, the GO class, enrichment, the enrichment p-values, and enrichment FDR q-values for genes showing the most differential methylation in t(1,11) carriers compared to non-carriers. Enrichment is defined as (b/n) / (B/N), where b = the number of genes at the top of the p-value ranked list that is associated with a given GO term, n = the number of genes at the top of the p-value-ranked gene list, B = the total number of genes associated with a given GO term, and N = the total number of genes.

Gene(s)	Feature(s)	Region	Probes	DMR *p*-value
TNXB	Body	Chr6:32063516-32065113	51	2.46×10^{-13}
NA	IGR	Chr3:196704439-196707088	5	7.89×10^{-10}
C11orf75	TSS1500	Chr11:93277097-93277255	3	1.67×10^{-9}
NA;*PRRT1*	IGR, 3'UTR	Chr6:32115866-32116728	14	2.60×10^{-9}
RNF5P1;AGPAT1	TSS1500, TSS200	Chr6:32145233-32145902	20	6.26×10^{-9}
GABRG1	TSS1500, TSS200, 5'UTR	Chr4:46125801-46126455	7	7.27×10^{-9}
KRTAP5-9	TSS1500, TSS200, 5'UTR	Chr11:71259142-71259846	5	3.17×10^{-8}
CYP2E1	Body	Chr10:135343047-135343426	3	5.93×10^{-8}
XRRA1	TSS200	Chr11:74660246-74660274	4	6.07×10^{-8}
RHOD	3'UTR	Chr11:66839183-66839543	3	8.99×10^{-8}
TUBGCP5	TSS1500, TSS200	Chr15:22833108-22833236	3	1.58×10^{-7}
TRAK1	TSS1500, Body, 1stExon	Chr3:42201087-42202130	6	1.64×10^{-7}
PSMB8	3'UTR, Body	Chr6:32807898-32810304	22	3.32×10^{-7}
NA	IGR	Chr1:209526301-209528771	3	3.46×10^{-7}
NA	IGR	Chr6:159359910-159360236	3	1.48×10^{-6}
RTKN	TSS1500	Chr2:74669347-74669415	4	1.94×10^{-6}
NA; *KLHL30*	IGR, TSS1500, TSS200	Chr2:239043942-239047460	9	2.67×10^{-6}
PCSK6	Body	Chr15:101991031-101992367	5	2.8×10^{-6}
MIR663	TSS1500	Chr20:26190290-26190418	3	3.66×10^{-6}
ZNF385D	Body, 1stExon	Chr3:21791767-21792729	4	3.82×10^{-6}
TNF	1stExon	Chr6:31543557-31543767	6	4.05×10^{-6}
BNIP3	Body	Chr10:133793398-133793556	3	9.21×10^{-6}
TMEM131	Body	Chr2:98377310-98378420	3	9.58×10^{-6}
LTA	TSS200, 5'UTR, 1stExon	Chr6:31540040-31540705	11	9.59×10^{-6}
NA	IGR	Chr17:41380728-41383399	5	9.98×10^{-6}
MIXL1	TSS1500	Chr1:226411005-226411085	4	1.06×10^{-5}
C17orf98	Body, 1stExon	Chr17:36997274-36997566	3	1.3×10^{-5}
FAM83A	Body	Chr8:124217614-124217906	3	1.42×10^{-5}
SP140	TSS200, 5'UTR, Body	Chr2:231090159-231091121	5	1.56×10^{-5}
MEST	5'UTR	Chr7:130131819-130131937	8	3×10^{-5}
MOV10L1	TSS200, Body	Chr22:50584735-50585710	7	3.02×10^{-5}
NA	IGR	Chr5:101117938-101120956	5	3.37×10^{-5}
NA	IGR	Chr2:54934730-54938012	4	3.42×10^{-5}

LOC399815	TSS200	Chr10:124638974-124638992	3	3.43×10^{-5}
STX6	TSS200	Chr1:180992101-180992126	3	3.44×10^{-5}
NA	IGR	Chr7:156888064-156890971	5	3.65×10^{-5}
NA	IGR	Chr14:54814555-54817362	4	5.68×10^{-5}
NA	IGR	Chr7:73001195-73003686	3	6.35×10^{-5}
NA	IGR	Chr4:77341749-77342075	3	6.66×10^{-5}
TDH	Body	Chr8:11203736-11204163	4	7.48×10^{-5}
SMYD2	Body	Chr1:214476582-214477761	3	9.03×10^{-5}
IGSF9B	Body	Chr11:133820356-133821733	4	9.05×10^{-5}
TAP1	Body	Chr6:32819956-32820360	7	9.06×10^{-5}
NA	IGR	Chr1:39281563-39282050	4	9.11×10^{-5}
NA	IGR	Chr10:134898718-134899190	3	9.24×10^{-5}
NA	IGR	Chr11:123939376-123941869	3	0.0001
NA	IGR	Chr1:147799913-147804738	9	0.0001
HLA-DPA1	Body, 3'UTR	Chr6:33033283-33037541	16	0.0001
MIR548N	Body	Chr2:179387372-179388545	3	0.0001
LIME1	Body	Chr20:62369366-62369583	3	0.0001
NA	IGR	Chr7:1208305-1210932	3	0.0001
VSTM1	TSS200, Body, 1stExon	Chr19:54566357-54567319	3	0.0001
NA	IGR	Chr5:61027075-61030044	6	0.0001
ZNF677	TSS200	Chr19:53758279-53758315	3	0.0001
NA	IGR	Chr11:8290652-8290978	3	0.0002
ACP5	TSS1500	Chr19:11689791-11689944	5	0.0002
PSKH2	TSS1500, TSS200	Chr8:87082001-87082129	5	0.0002
ATP8A2	Body	Chr13:26586108-26586400	3	0.0002
VAX2	TSS1500	Chr2:71126945-71127001	3	0.0002
LRP1B	TSS200	Chr2:142889366-142889402	3	0.0003
C2orf54	TSS1500	Chr2:241836300-241836518	3	0.0003
KCNIP1; *KCNMB1*	TSS200, Body	Chr5:169815897-169816859	4	0.0003
NA	IGR	Chr8:832849-833211	4	0.0003
C6orf48	5'UTR	Chr6:31802972-31803220	9	0.0004
NHEDC1	TSS200, 1stExon	Chr4:103940811-103940897	3	0.0004
HEATR2	Body, 3'UTR	Chr7:824351-826059	6	0.0004
NA	IGR	Chr10:115860117-115860473	3	0.0004

OR2L13	1stExon	Chr1:248100542-248100657	4	0.0005
RPTOR	Body	Chr17:78809217-78810354	3	0.0005
ADARB2	Body	Chr10:1452489-1453586	3	0.0005
KLHL29	Body	Chr2:23885619-23886838	3	0.0005
NA	IGR	Chr6:33870717-33874881	16	0.0006
C2CD4D	Body	Chr1:151810808-151810978	4	0.0006
KATNAL2	5'UTR	Chr18:44561939-44562173	5	0.0006
NA	IGR	Chr1:46632418-46632744	4	0.0007
NA	IGR	Chr1:117316713-117319422	6	0.0007
RHOU	Body	Chr1:228872106-228872264	3	0.0007
ADAMTS2	Body	Chr5:178594504-178594662	3	0.0007
NFE2L1	TSS1500	Chr17:46124661-46124789	3	0.0007
TBCD	Body	Chr17:80759197-80760159	3	0.0007
RP1	Body	Chr8:55533808-55534034	4	0.0007
DPYSL3	Body	Chr5:146832152-146832444	5	0.0008
DZIP1L	TSS1500	Chr3:137834643-137834771	3	0.0009
NA	IGR	Chr12:29302148-29302314	3	0.0009
NA	IGR	Chr12:114916775-114919883	6	0.0010
MSX1	Body	Chr4:4864284-4864576	6	0.0010
NA	IGR	Chr6:32848233-32852184	3	0.0011
MAPT	1stExon	Chr17:43971863-43971971	4	0.0011
ERMAP	5'UTR, Body	Chr1:43295986-43296972	4	0.0012
TRIM10	3'UTR, Body	Chr6:30118914-30123074	24	0.0012
NA; *RPRM*	IGR, 3'UTR	Chr2:154333734-154334094	5	0.0013
NA	IGR	Chr1:160950485-160953893	5	0.0013
NA	IGR	Chr10:133528532-133530912	4	0.0016
HOXC11	3'UTR	Chr12:54369487-54373005	6	0.0017
TSPAN19;LRRIQ1	TSS200, 5'UTR	Chr12:85430071-85430601	8	0.0018
TRIM15	3'UTR	Chr6:30140125-30140546	8	0.0019
PRICKLE1	Body, 3'UTR	Chr12:42850912-42854914	7	0.0019
STAT3	Body	Chr17:40489088-40490266	5	0.0021
NA; *COX19*	IGR, 3'UTR	Chr7:1001886-1005509	9	0.0022
NA	IGR	Chr8:53325115-53327858	8	0.0023
S100A13; *S100A14*	TSS1500, 3'UTR	Chr1:153589312-153593314	5	0.0036

MICB	Body	Chr6:31466095-31466266	4	0.0039
NA	IGR	Chr6:30094973-30095632	25	0.0040
SLC6A12	TSS200, 5'UTR	Chr12:322249-322914	9	0.0044
TNXB	Body	Chr6:32054900-32055225	8	0.0063
NA	IGR	Chr5:3102528-3106063	4	0.0064
TMCO3	Body	Chr13:114192835-114194543	11	0.0064
ASCL2	TSS1500	Chr11:2293053-2293275	9	0.0064
VARS2	Body	Chr6:30883736-30885444	14	0.0074
B3GALT4	1stExon	Chr6:33245445-33245846	20	0.0090
NOM1	Body	Chr7:156755232-156756616	6	0.0092
ALPL	5'UTR	Chr1:21877057-21877844	6	0.0105
HLA-DPA1	Body	Chr6:33040054-33041250	8	0.0121
EHMT2	Body	Chr6:31858853-31861146	20	0.0124
NA	IGR	Chr12:131700432-131703053	5	0.0166
KLC2	3'UTR	Chr11:66034892-66035327	14	0.0169
CNTN4	Body	Chr3:3079716-3080819	4	0.0224
GNL1	3'UTR	Chr6:30507466-30511643	15	0.0289
PRDM16	Body	Chr1:3209038-3210391	5	0.0380
HCP5	TSS1500, TSS200, 1stExon, Body, 3'UTR	Chr6:31429311-31433970	18	0.0392
NA	IGR	Chr5:170762452-170765146	9	0.0408
ARHGAP25	TSS1500, 5'UTR, Body	Chr2:69001334-69002335	10	0.0428
HSPA1L	5'UTR, Body	Chr6:31778918-31782134	19	0.0498

Supplementary Table 5 Summary of t(1;11)-associated DMRs identified by the probe lasso algorithm

Summary of the genes (if applicable), genomic features, hg19 genomic coordinates, number of probes and FDR-corrected p-value (q-value) associated with each DMR. “NA” in the “Gene” column represents intergenic regions (i.e. regions not annotated to a RefSeq gene). Genomic features are coded “IGR” for intergenic regions, “TSS200” and “TSS1500” for probes occurring within 200 and 1500 of a gene’s transcription start site, respectively; “5’UTR” and “3’UTR” for probes occurring within a gene’s 5’ and 3’ untranslated region, respectively, “1stExon” for probes occurring within the first exon of a gene, and “Body” for probes occurring within the gene body.

	t(1;11) Non-carriers	*t(1;11) Carriers*
Unaffected	18	
Recurrent MDD	2	3
Single episode MDD	2	3
Single episode MDD + generalised anxiety	1	
Generalised anxiety	1	
Conduct disorder (adolescent)		2
Single episode MDD + Conduct disorder (adolescent)		1
Cyclothymia		3
Bipolar Disorder I		2
Schizophrenia		2
Schizoaffective disorder		1

Supplementary Table 6 Diagnosis counts within each group

Shown is a summary of psychiatric diagnoses in the family and the number of t(1;11) carriers and non-carriers with a given diagnosis.
