# Supplementary material for: Altered DNA methylation associated with a translocation linked to major mental illness
Source: NPJ Schizophr. 2018 Mar 19;4:5. doi: 10.1038/s41537-018-0047-7 (PMC5859082; doi:10.1038/s41537-018-0047-7)
Supplement: Supplementary file 2 — Supplementary Figures [file 41537_2018_47_MOESM2_ESM.pdf]

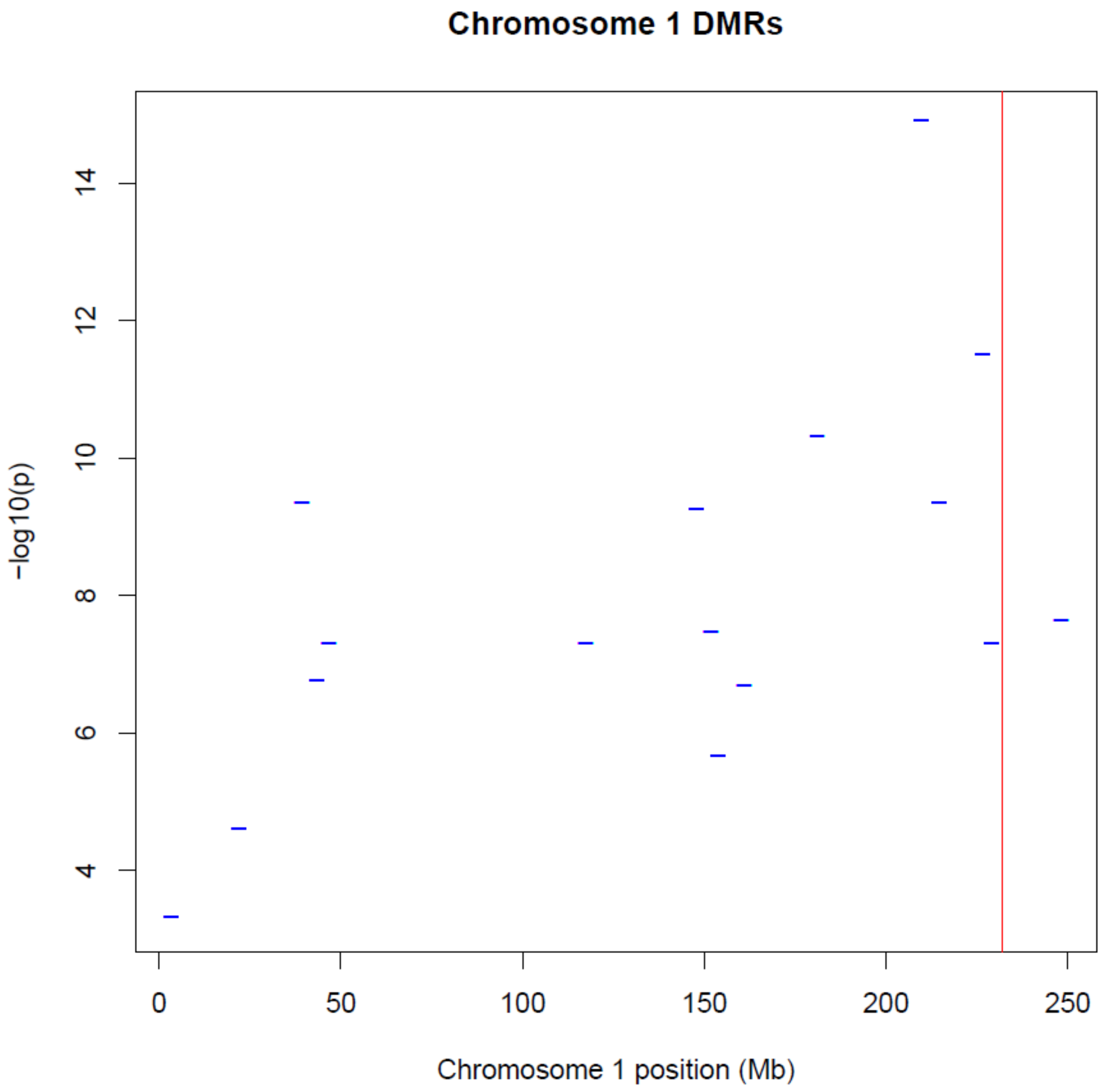

Supplementary Figure 1 DMRs identified on chromosome 1
DMRs identified between t(1;11) carriers and non-carriers on chromosome 1. Chromosomal position in megabases (Mb) is shown along the X-axis while –log10 p-value is shown along the Y-axis. The red vertical line represents the t(1;11) breakpoint on chromosome 1.

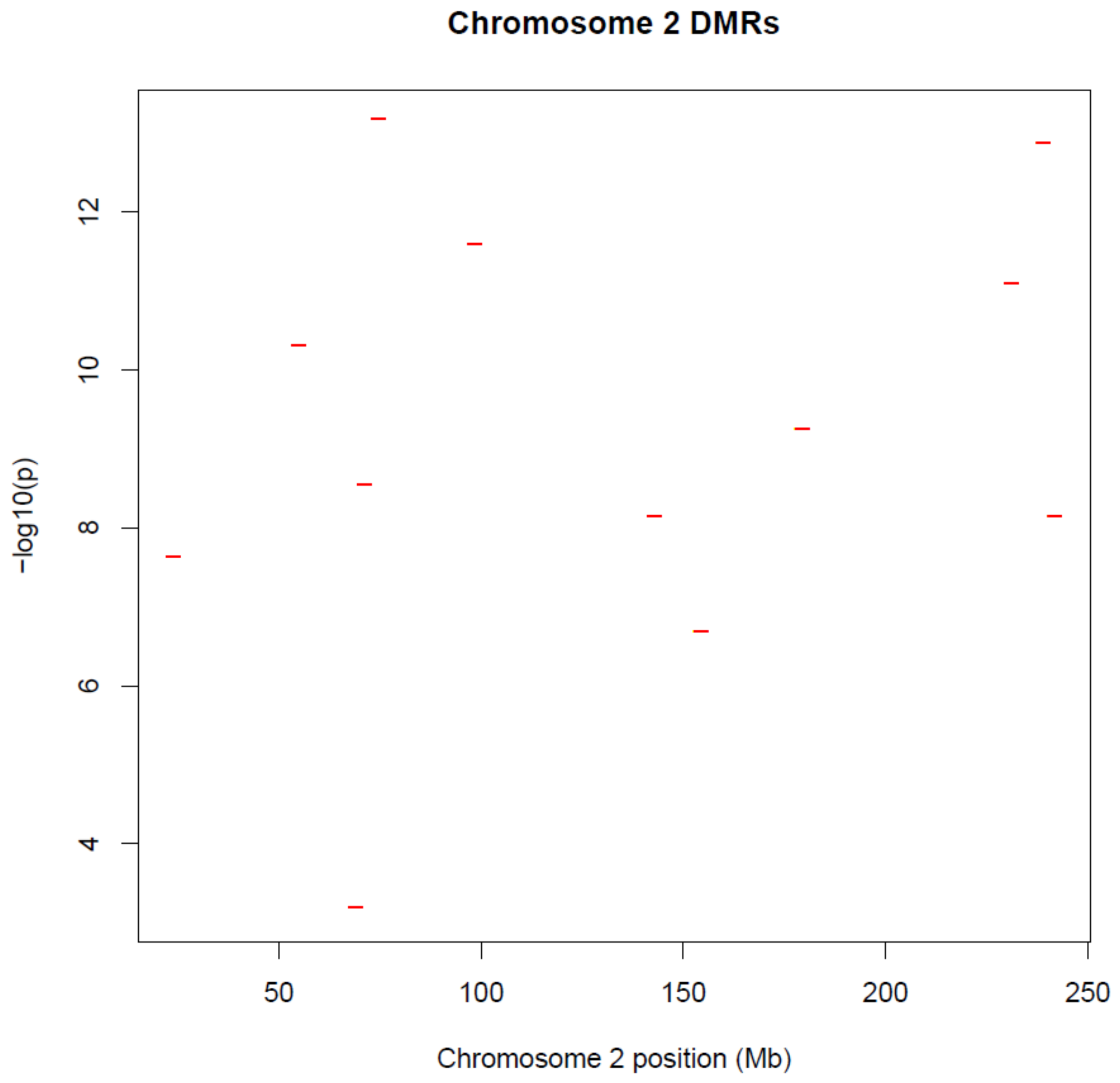

Supplementary Figure 2 DMRs identified on chromosome 2
DMRs identified between t(1;11) carriers and non-carriers on chromosome 2. Chromosomal position in megabases (Mb) is shown along the X-axis while –log10 p-value is shown along the Y-axis.

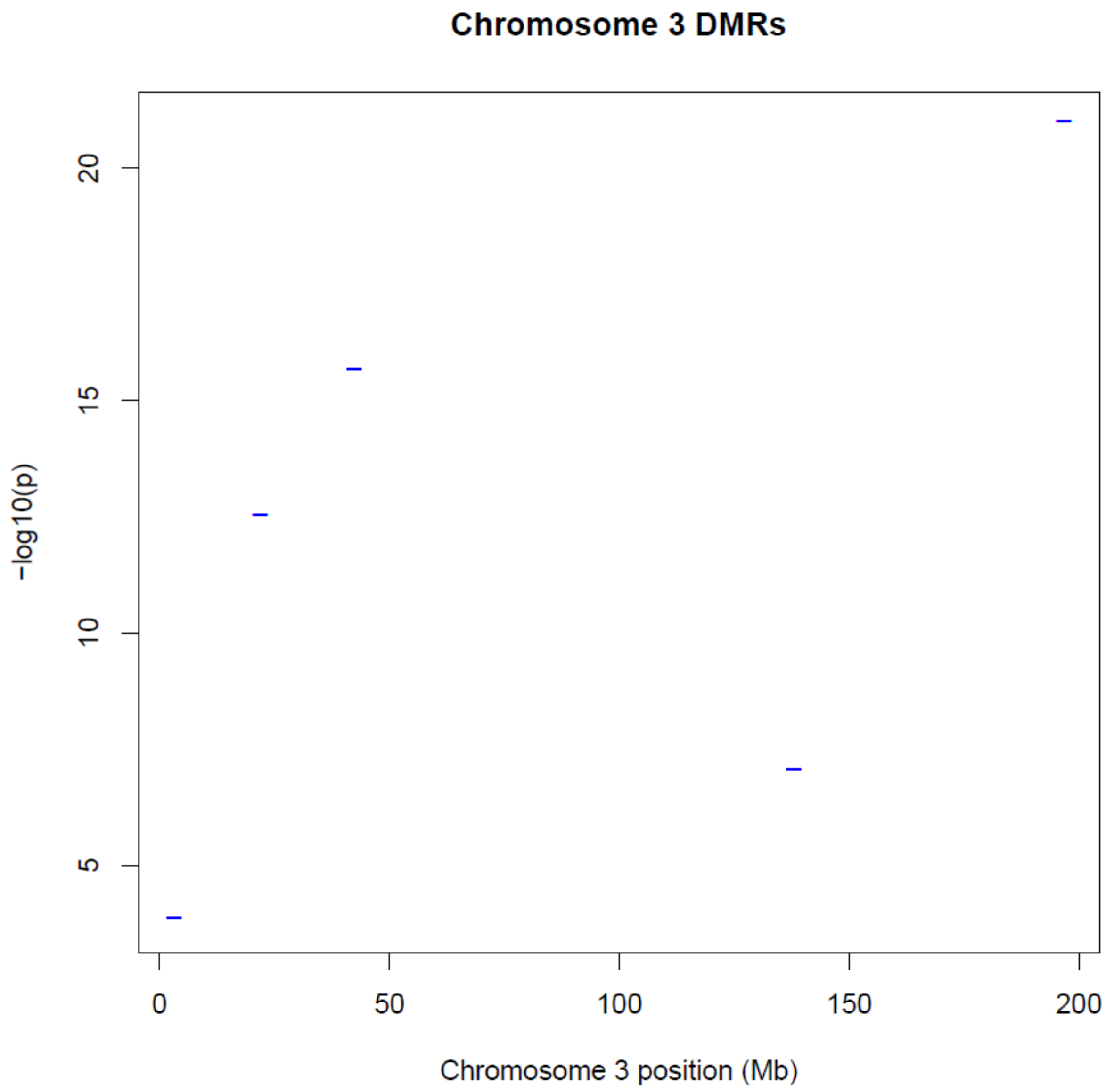

Supplementary Figure 3 DMRs identified on chromosome 3
DMRs identified between t(1;11) carriers and non-carriers on chromosome 3. Chromosomal position in megabases (Mb) is shown along the X-axis while –log10 p-value is shown along the Y-axis.

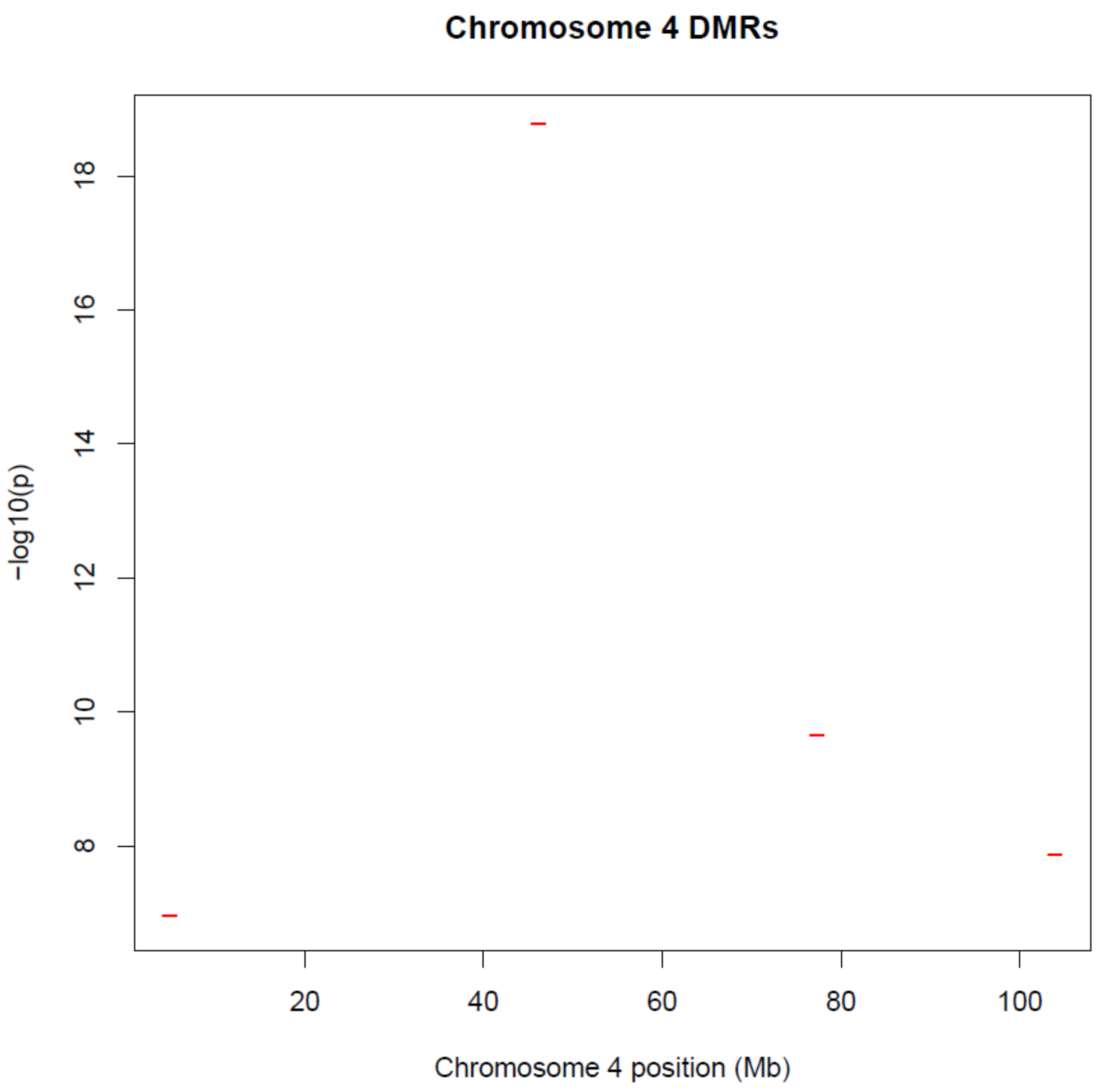

Supplementary Figure 4 DMRs identified on chromosome 4
DMRs identified between t(1;11) carriers and non-carriers on chromosome 4. Chromosomal position in megabases (Mb) is shown along the X-axis while –log10 p-value is shown along the Y-axis.

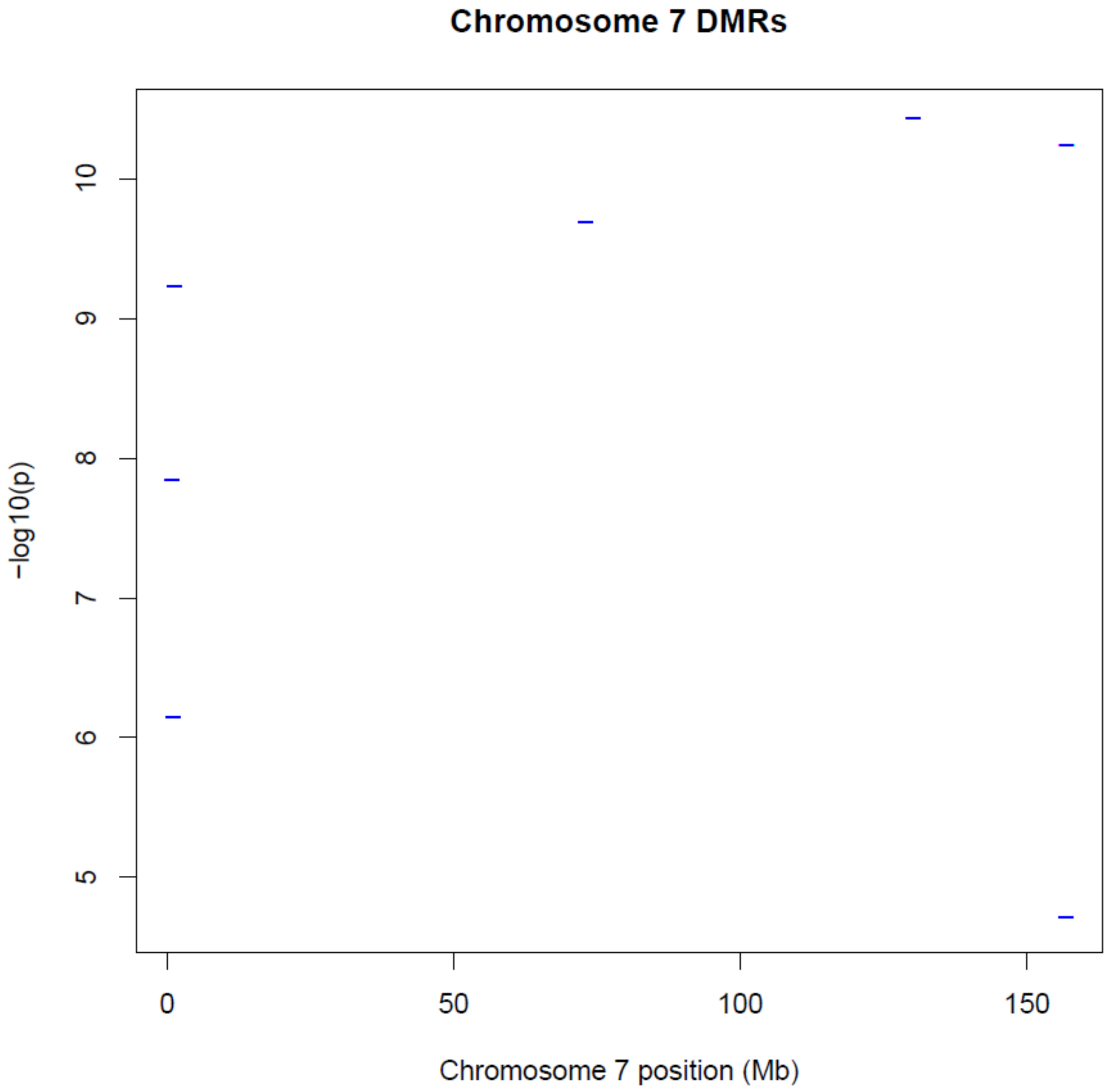

Supplementary Figure 5 DMRs identified on chromosome 7
DMRs identified between t(1;11) carriers and non-carriers on chromosome 7. Chromosomal position in megabases (Mb) is shown along the X-axis while –log10 p-value is shown along the Y-axis.

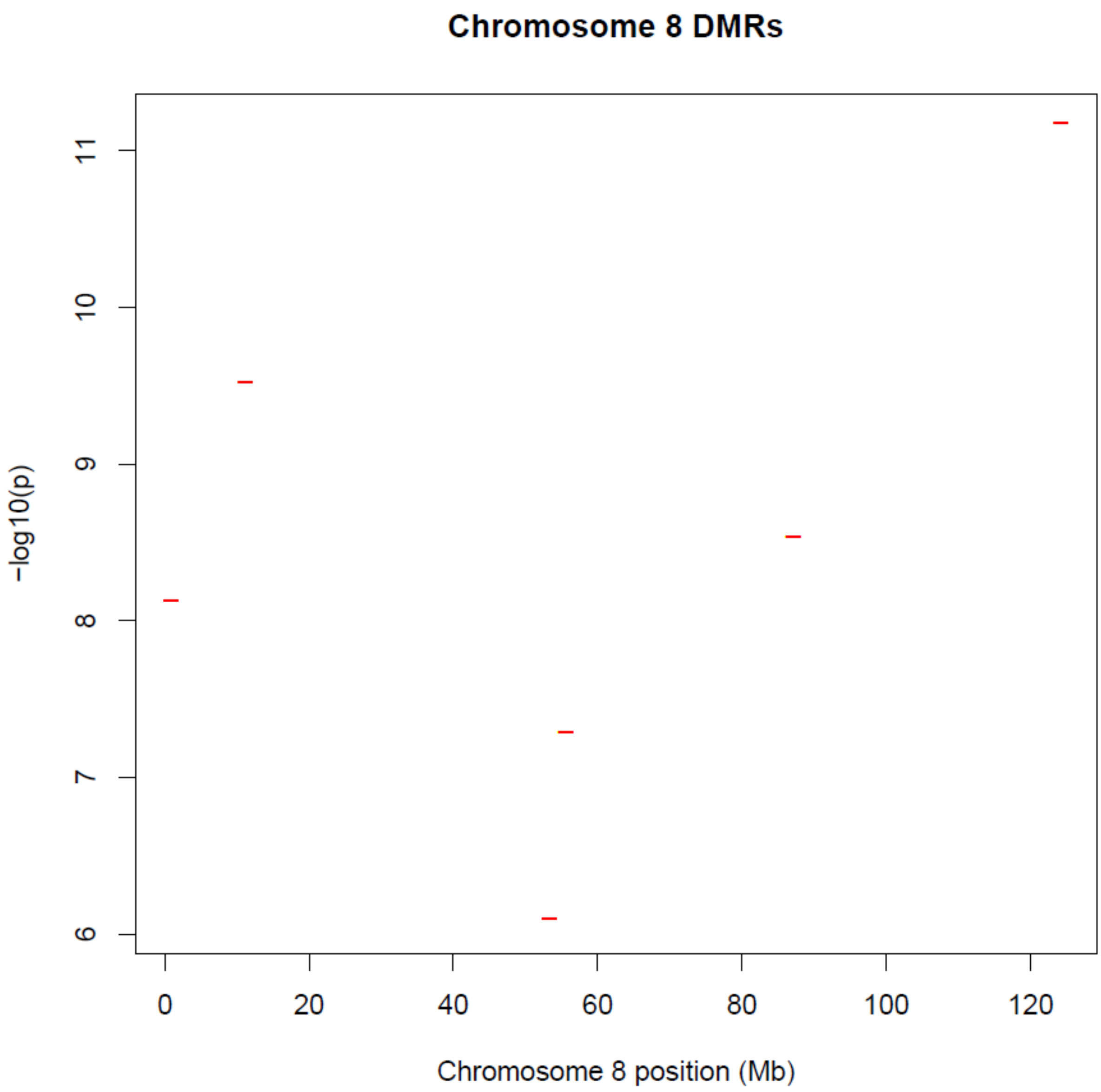

Supplementary Figure 6 DMRs identified on chromosome 8
DMRs identified between t(1;11) carriers and non-carriers on chromosome 8. Chromosomal position in megabases (Mb) is shown along the X-axis while –log10 p-value is shown along the Y-axis.

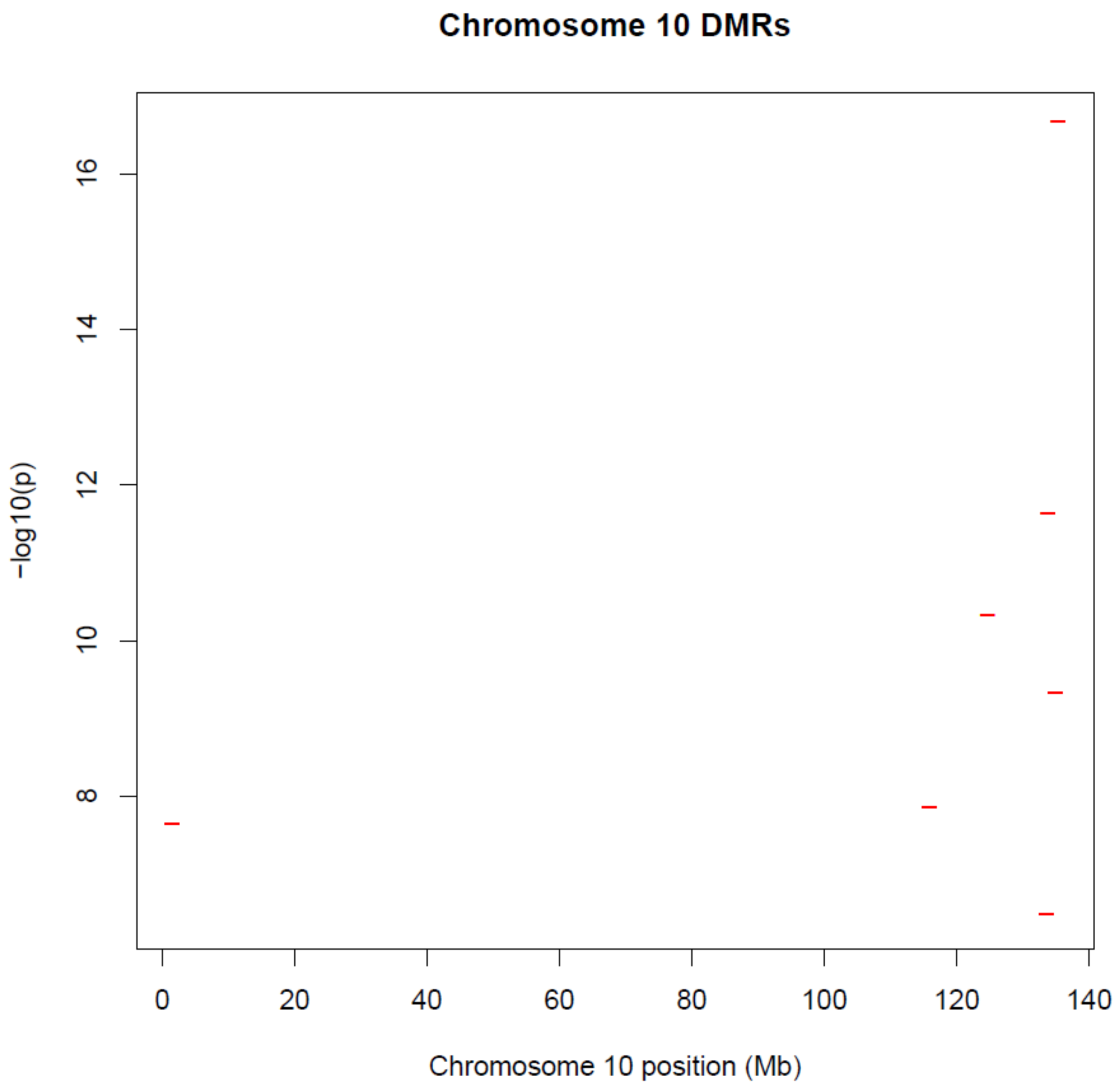

Supplementary Figure 7 DMRs identified on chromosome 10
DMRs identified between t(1;11) carriers and non-carriers on chromosome 10. Chromosomal position in megabases (Mb) is shown along the X-axis while –log10 p-value is shown along the Y-axis.

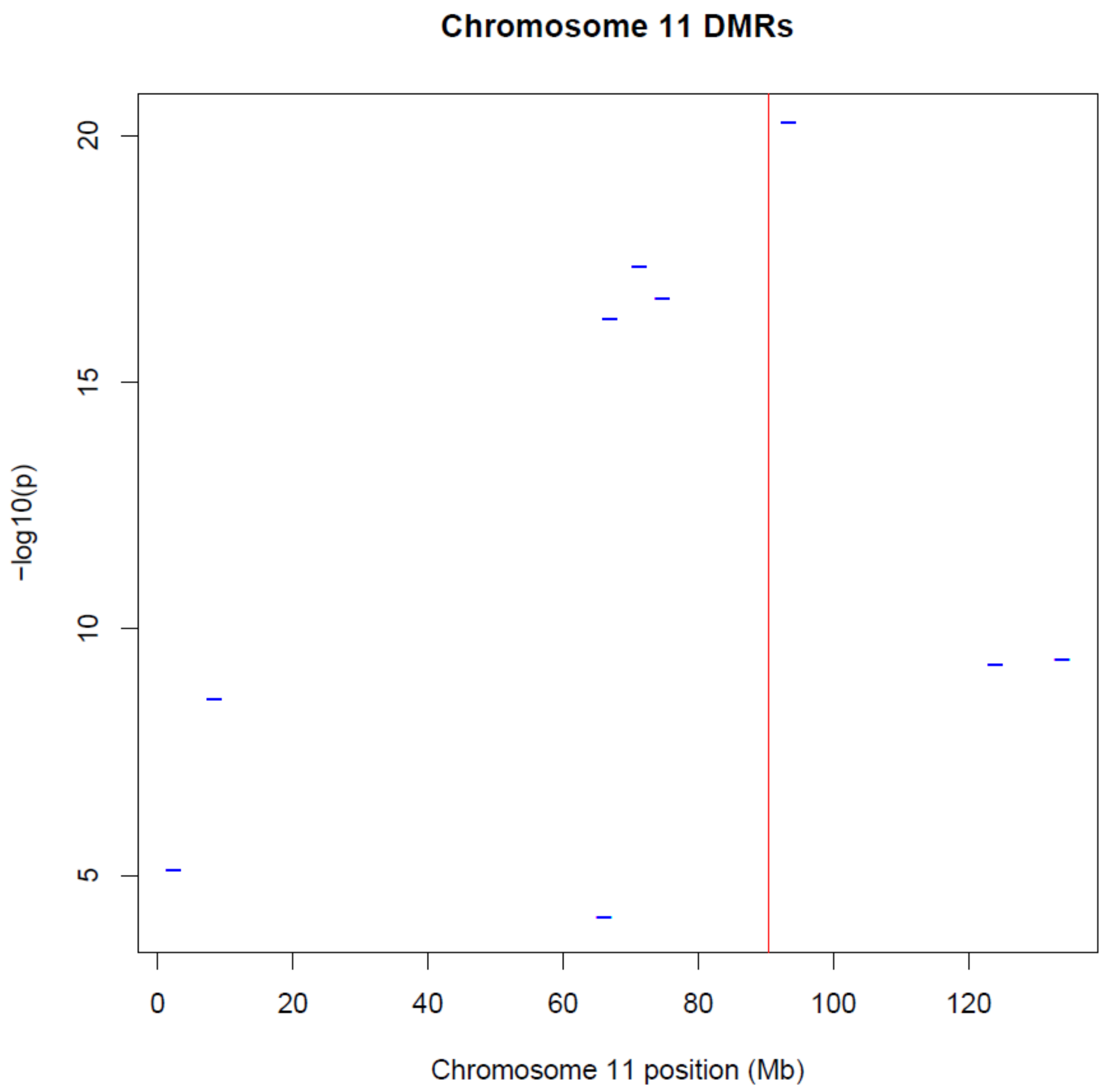

Supplementary Figure 8 DMRs identified on chromosome 11
DMRs identified between t(1;11) carriers and non-carriers on chromosome 11. Chromosomal position in megabases (Mb) is shown along the X-axis while –log10 p-value is shown along the Y-axis. The red vertical line represents the t(1;11) breakpoint on chromosome 11.

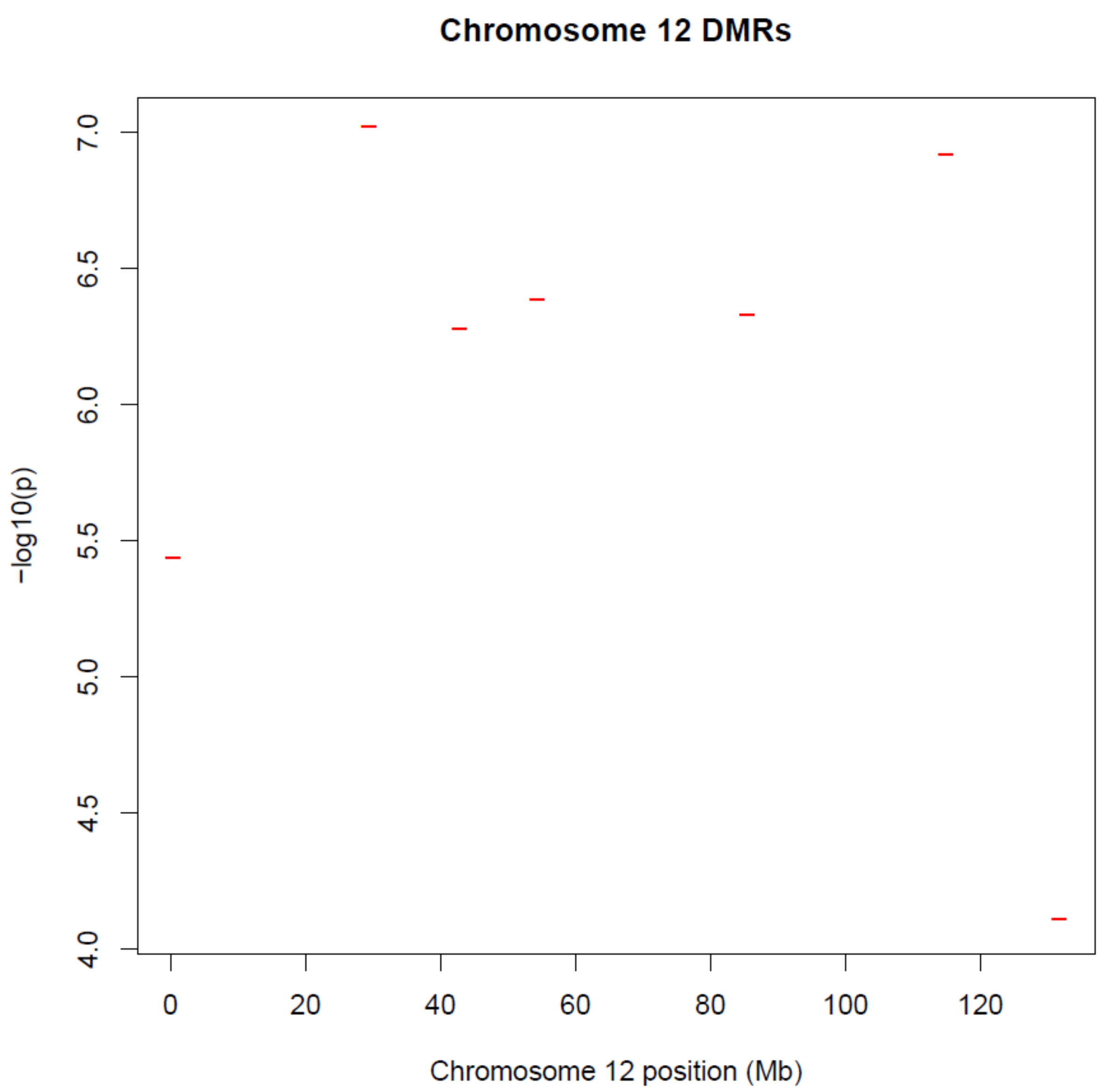

Supplementary Figure 9 DMRs identified on chromosome 12
DMRs identified between t(1;11) carriers and non-carriers on chromosome 12. Chromosomal position in megabases (Mb) is shown along the X-axis while –log10 p-value is shown along the Y-axis.

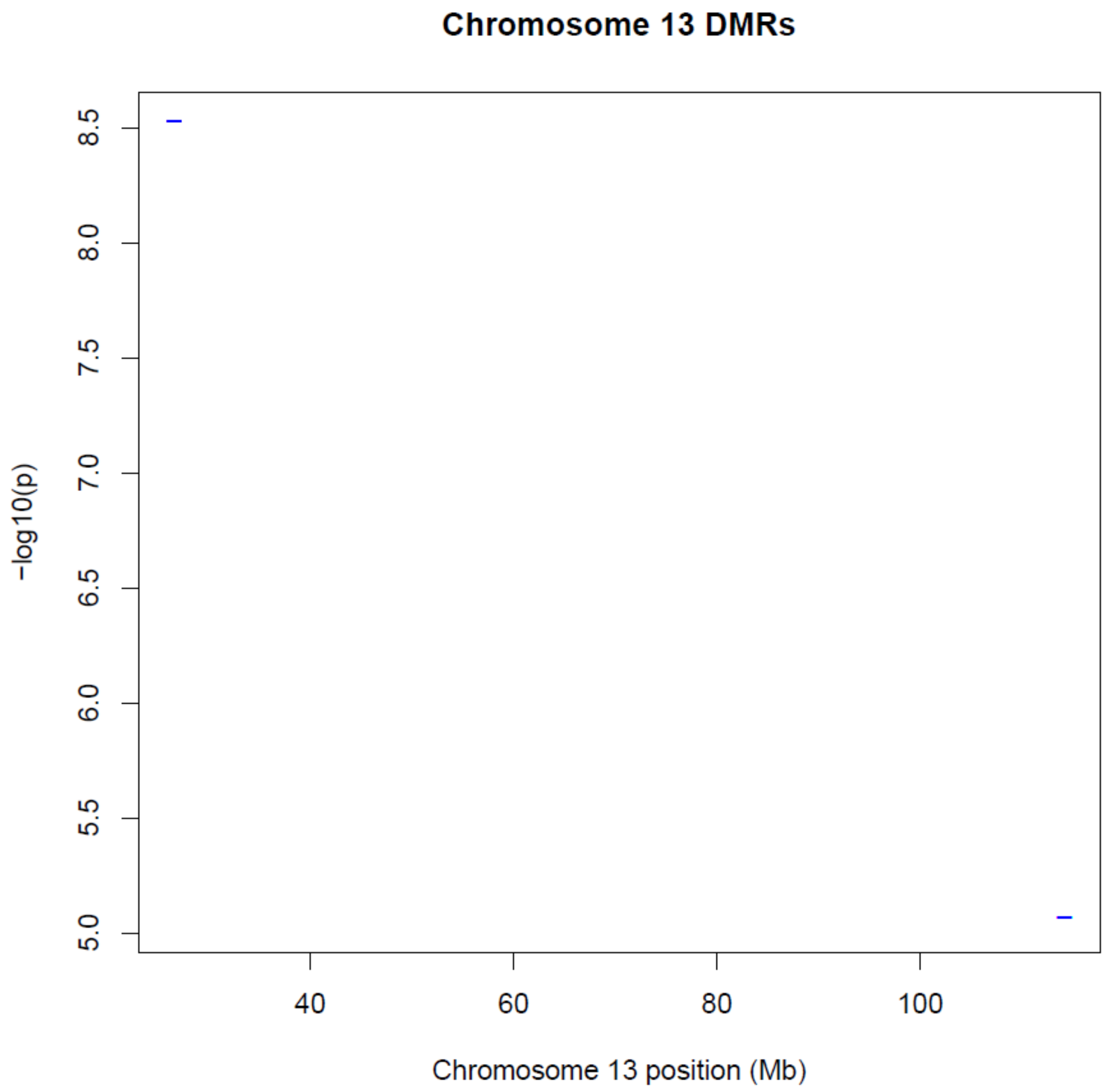

Supplementary Figure 10 DMRs identified on chromosome 13
DMRs identified between t(1;11) carriers and non-carriers on chromosome 13. Chromosomal position in megabases (Mb) is shown along the X-axis while –log10 p-value is shown along the Y-axis.

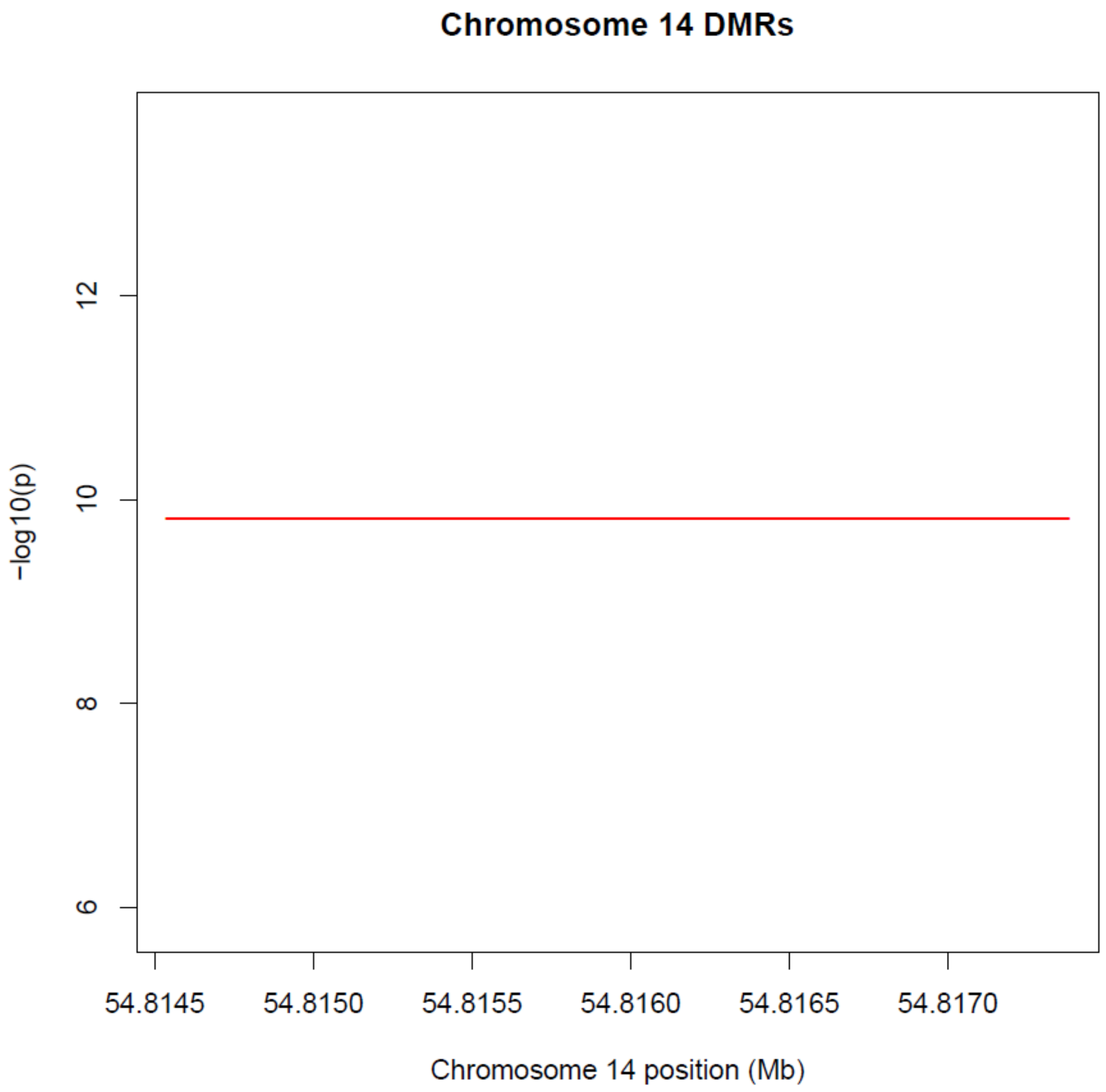

Supplementary Figure 11 DMR identified on chromosome 14
DMR identified between t(1;11) carriers and non-carriers on chromosome 14. Chromosomal position in megabases (Mb) is shown along the X-axis while –log10 p-value is shown along the Y-axis.

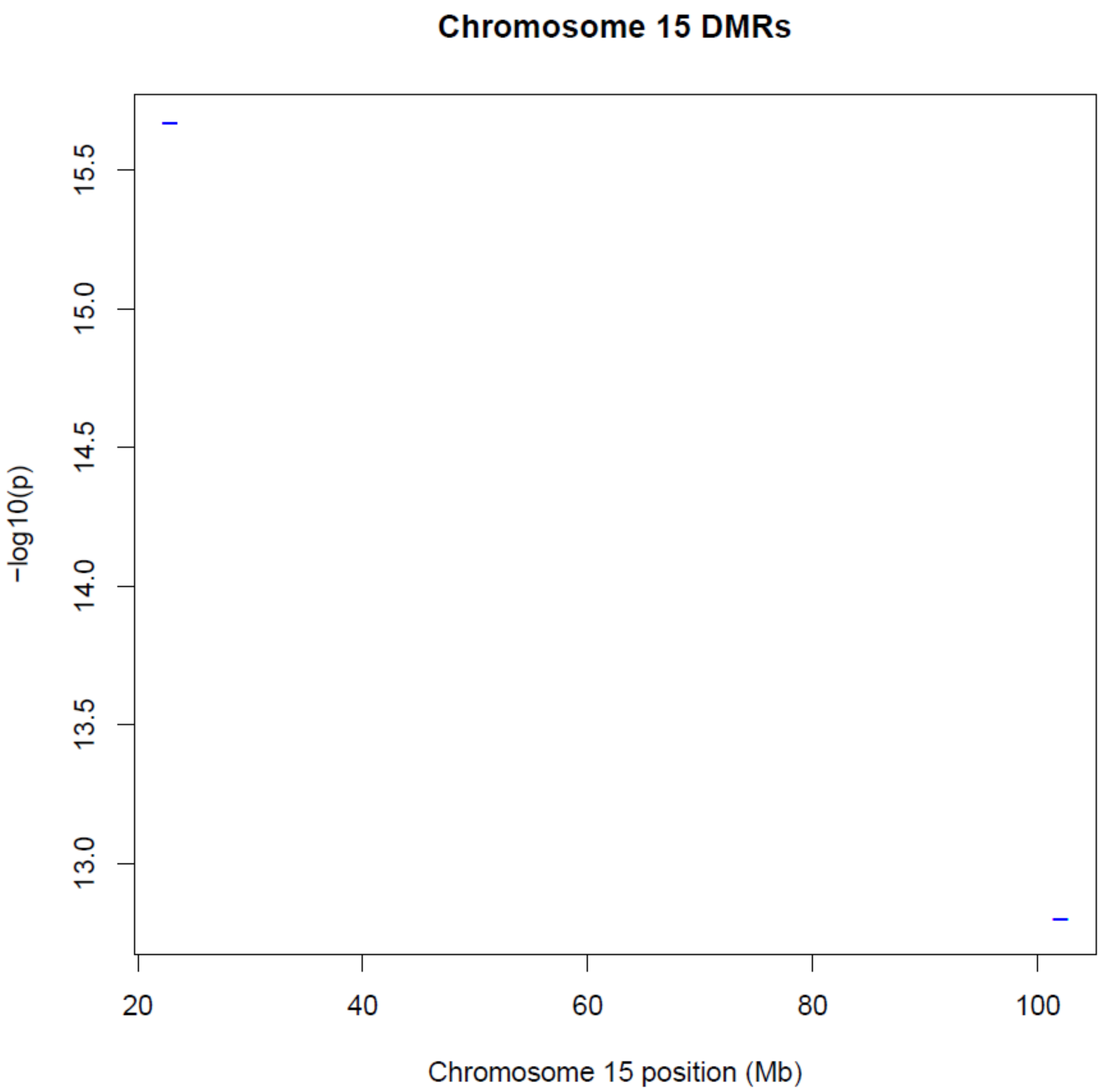

Supplementary Figure 12 DMRs identified on chromosome 15
DMRs identified between t(1;11) carriers and non-carriers on chromosome 15. Chromosomal position in megabases (Mb) is shown along the X-axis while –log10 p-value is shown along the Y-axis.

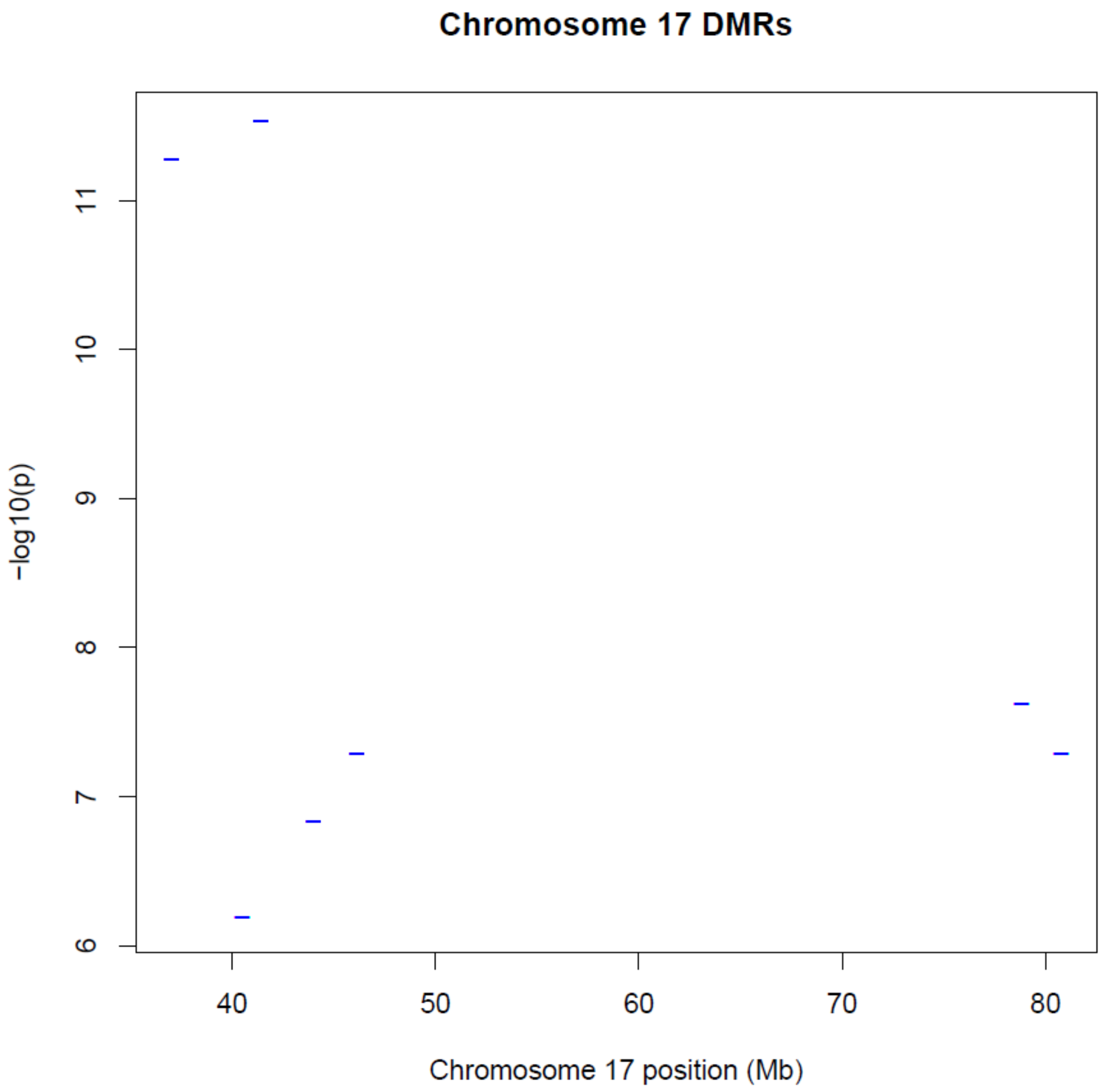

Supplementary Figure 13 DMRs identified on chromosome 17
DMRs identified between t(1;11) carriers and non-carriers on chromosome 17. Chromosomal position in megabases (Mb) is shown along the X-axis while –log10 p-value is shown along the Y-axis.

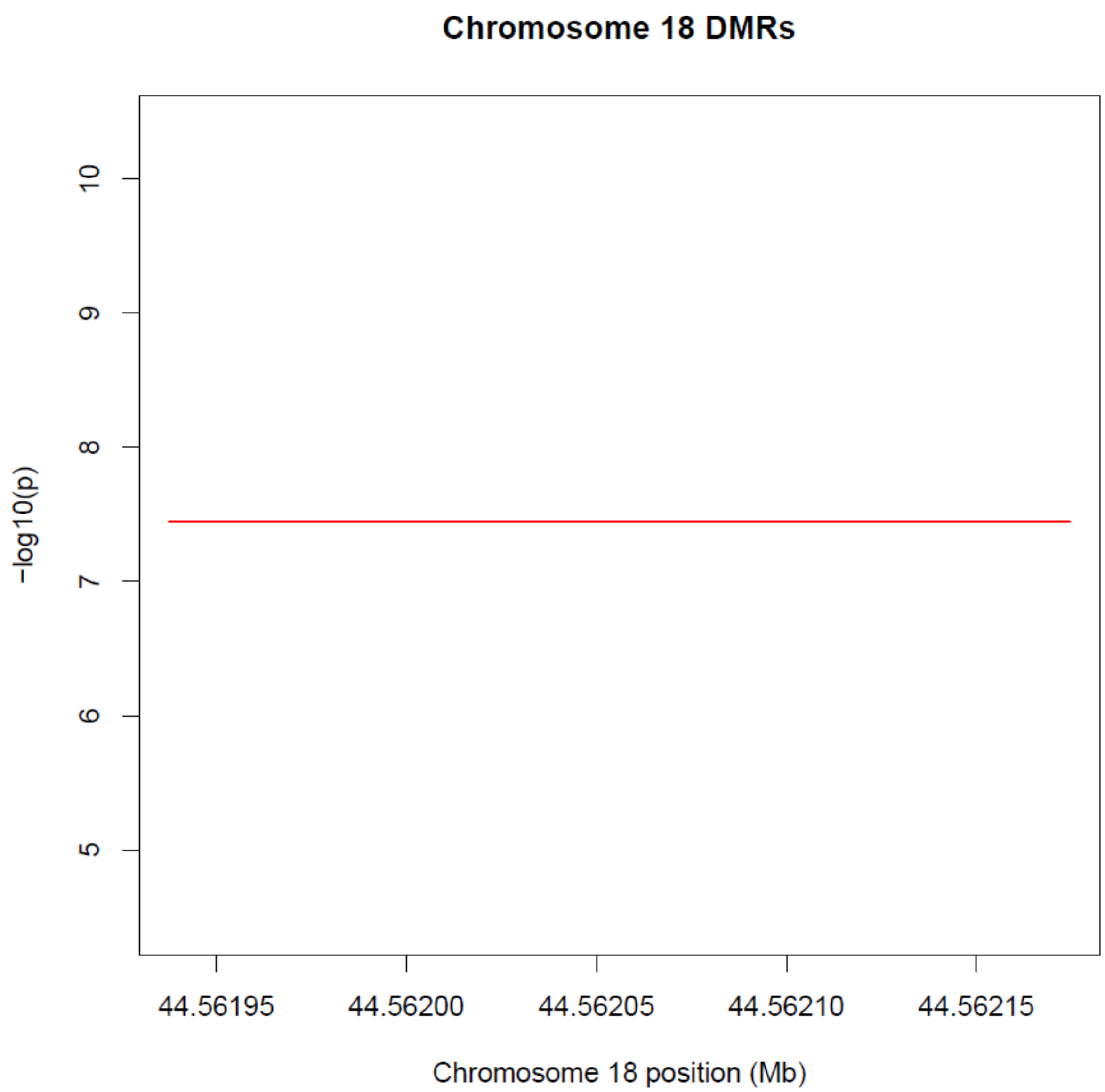

Supplementary Figure 14 DMR identified on chromosome 18
DMR identified between t(1;11) carriers and non-carriers on chromosome 18. Chromosomal position in megabases (Mb) is shown along the X-axis while –log10 p-value is shown along the Y-axis.

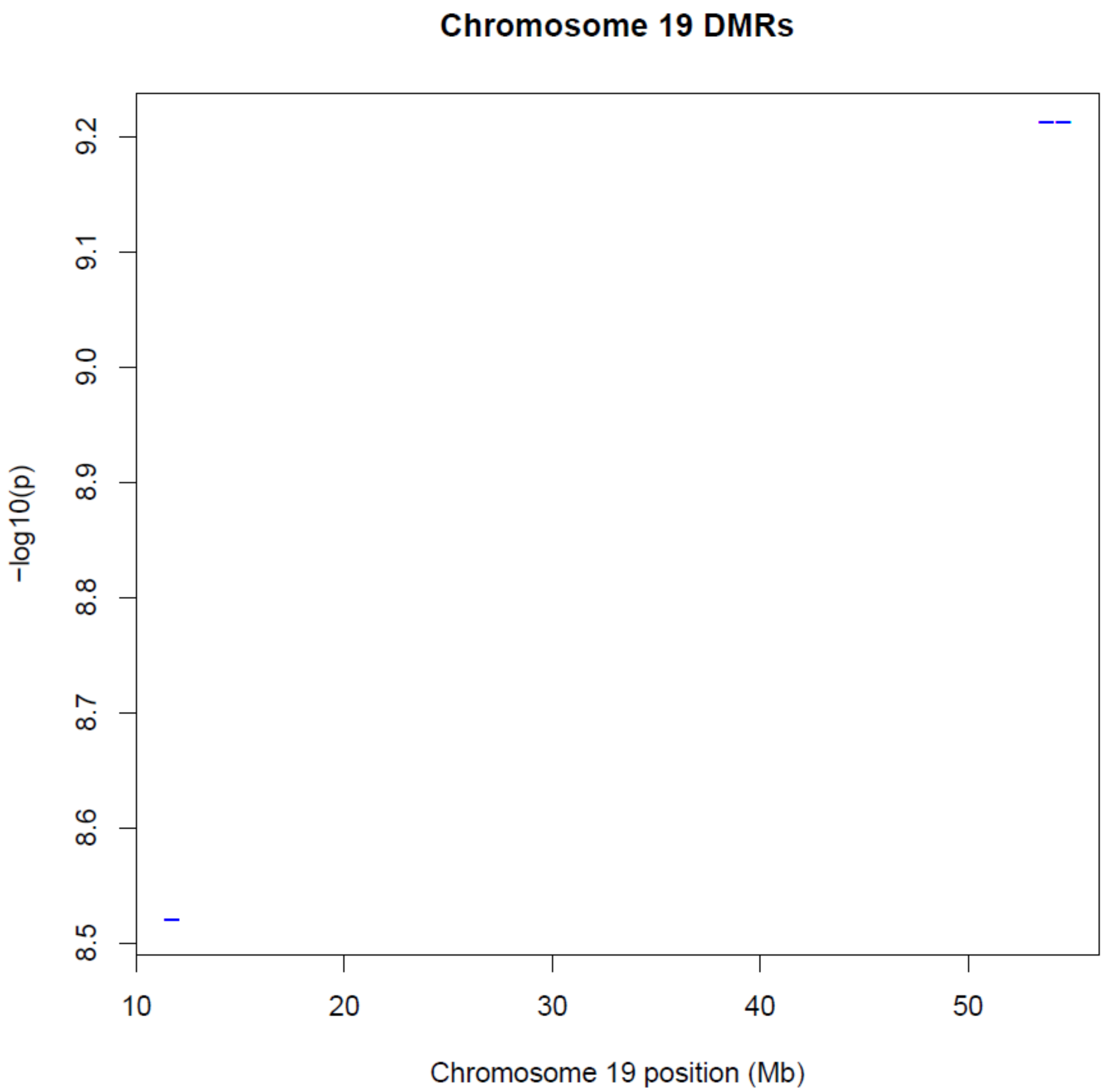

Supplementary Figure 15 DMRs identified on chromosome 19
DMRs identified between t(1;11) carriers and non-carriers on chromosome 19. Chromosomal position in megabases (Mb) is shown along the X-axis while –log10 p-value is shown along the Y-axis.

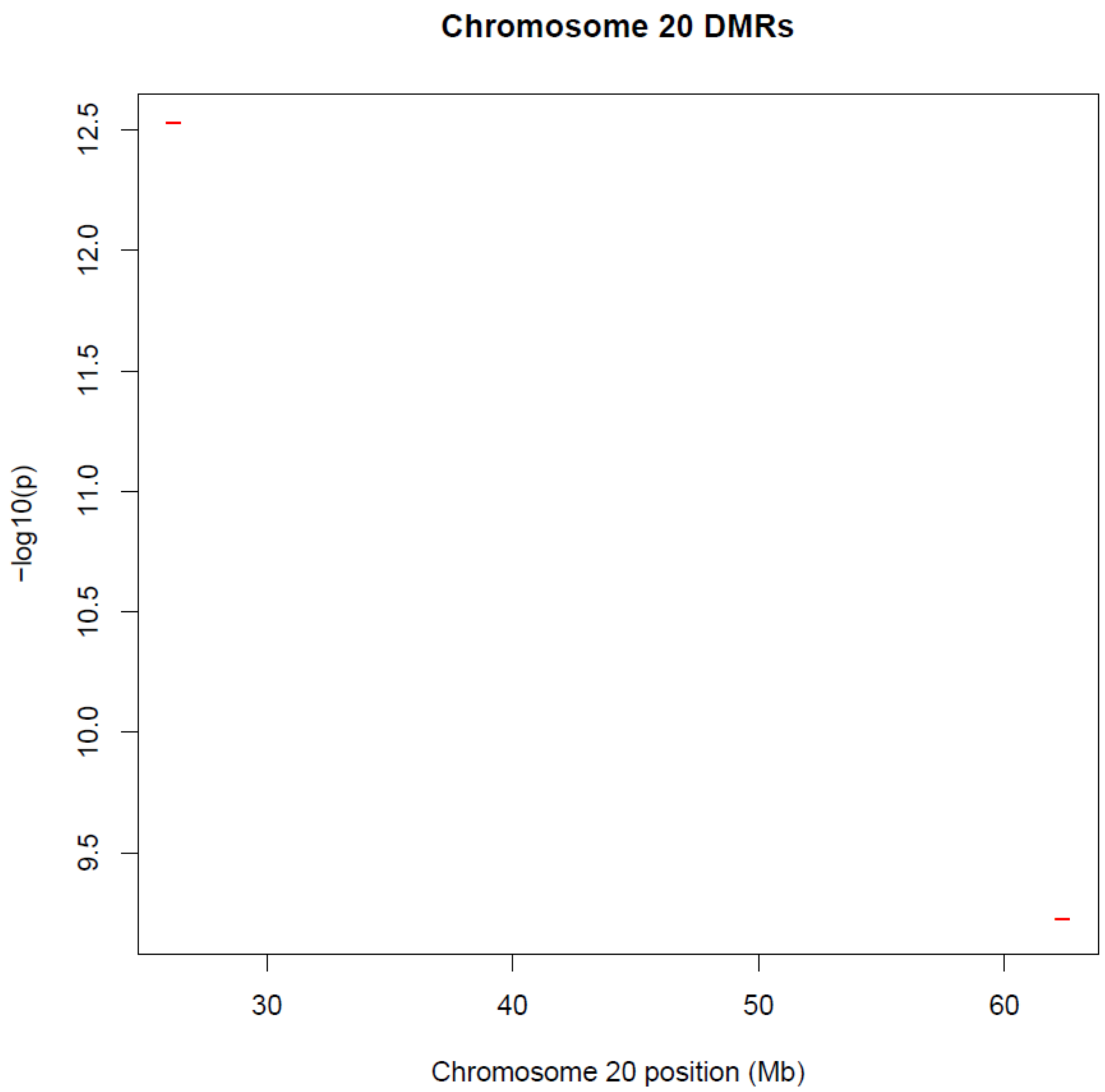

Supplementary Figure 16 DMRs identified on chromosome 20
DMRs identified between t(1;11) carriers and non-carriers on chromosome 20. Chromosomal position in megabases (Mb) is shown along the X-axis while –log10 p-value is shown along the Y-axis.

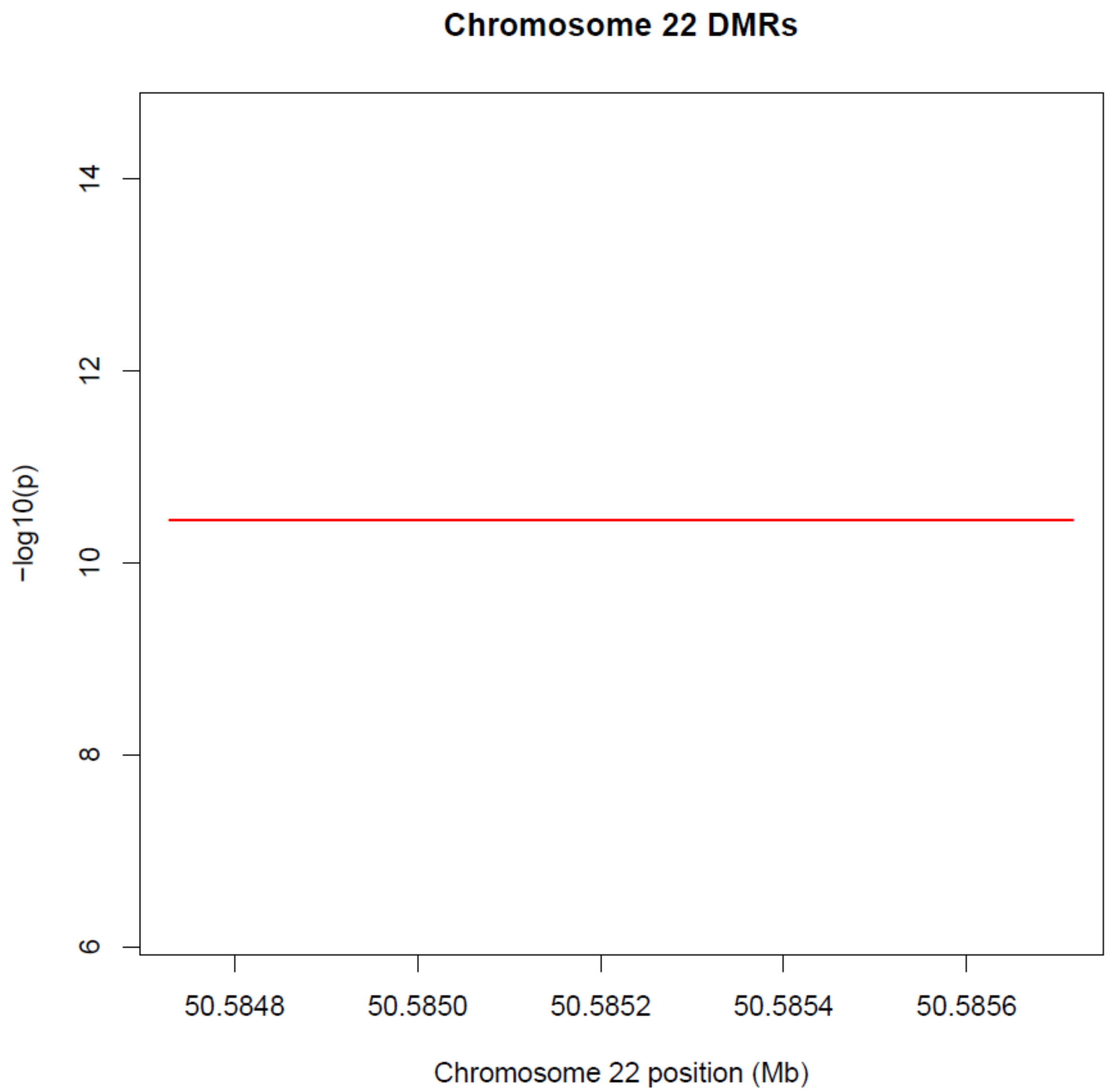

Supplementary Figure 17 DMRs identified on chromosome 22
DMRs identified between t(1;11) carriers and non-carriers on chromosome 22. Chromosomal position in megabases (Mb) is shown along the X-axis while –log10 p-value is shown along the Y-axis.

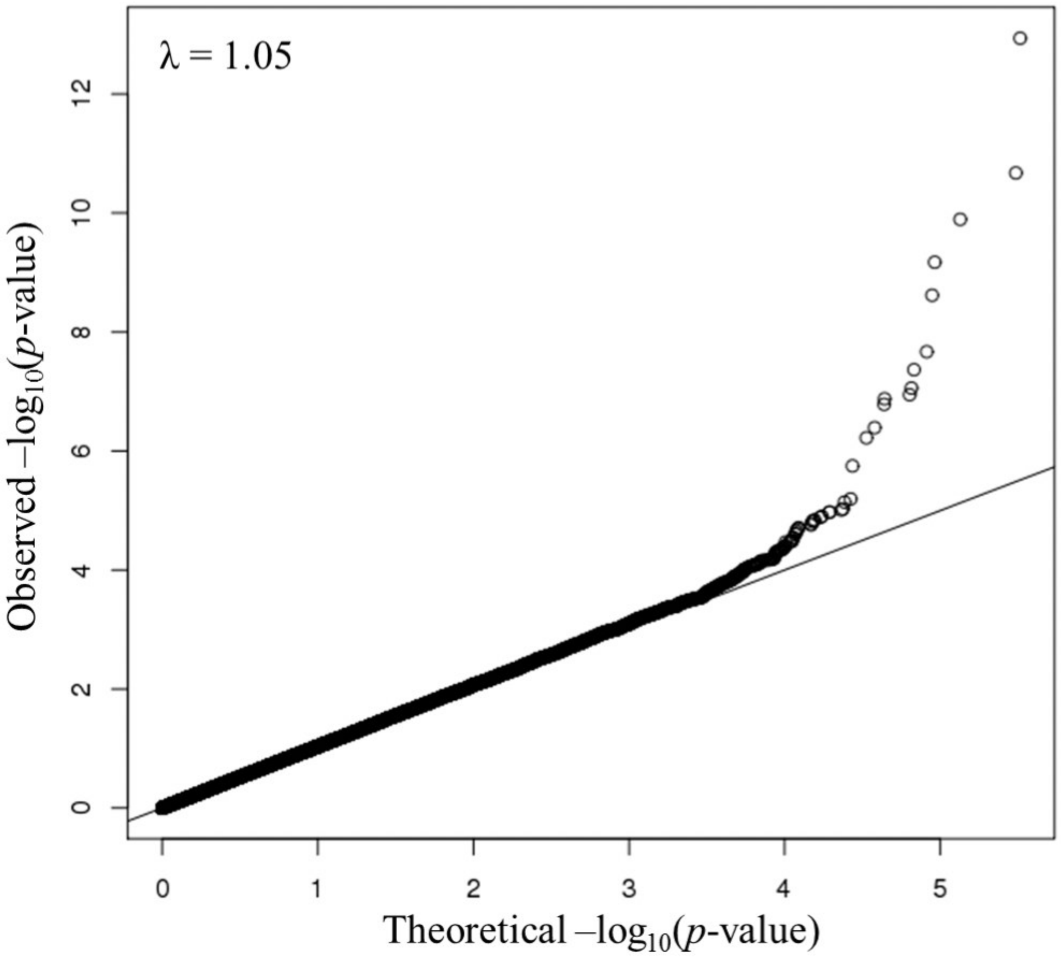

Supplementary Figure 18 Quantile-quantile plot showing observed vs. expected *p*-values for differential methylation between t(1;11) carriers and non-carriers

Shown are observed raw $-\log_{10}$ *p*-values (y-axis; circular points) plotted against the expected distribution of $-\log_{10}$ *p*-values under the null hypothesis (x-axis; solid diagonal line). The genomic inflation factor (λ) is presented in the upper-left corner of the plot.
